# The analysis of the oral DNA virome reveals which viruses are widespread and rare among healthy young adults in Valencia (Spain)

**DOI:** 10.1371/journal.pone.0191867

**Published:** 2018-02-08

**Authors:** Vicente Pérez-Brocal, Andrés Moya

**Affiliations:** 1 Genomics and Health, FISABIO-Public Health, Valencia, Spain; 2 CIBER Epidemiología y Salud Pública (CIBERESP), Madrid, Spain; 3 Integrative Systems Biology Institute (I2Sysbio) University of Valencia and Spanish Research Council (CSIC), Valencia, Spain; Institut Pasteur of Shanghai Chinese Academy of Sciences, CHINA

## Abstract

We have analysed oral wash samples from 72 healthy young adults in Valencia (Spain) for a metagenomic analysis through the construction of shotgun libraries and high-throughput-sequencing. The oral viral communities have been taxonomically characterised as well as and the gene content from the latter. The majority of viruses are found in few individuals, with single occurrences being the most widespread ones, whereas universally distributed viruses, while present, are relatively rare, with bacteriophages from families *Siphoviridae* and *Myoviridae*, and *Streptococcus* phages, as well as the eukaryotic viral family *Herpesviridae* amongst the most widespread viruses. No significant differences were found between females and males for either viruses and bacteria in abundance and alpha and beta diversity. The virome show similarities with other oral viromes previously reported for healthy individuals, suggesting the existence of a universal core of oral viruses, at least in the Western society, regardless of the geographical location.

## Introduction

The human microbiome, composed of the resident microbial communities hosted in or on the human body, largely outnumbers our own cells [1]. Those commensal communities include bacteria, archaea, viruses and eukaryotic organisms that interact with each other and with the human host. As a result, the microbiome confers the host effects such as resistance to colonisation by pathogens, provision of additional metabolic potential, support for host defence functions and other beneficial effects [2,3].

The gastrointestinal tract (GIT) is one of the most microbial-enriched environments in the human body, and one of the most surveyed ones. However, longitudinal differences are observed along the length of GIT, with the highest densities reached in the colon (up to 10^10−12^ cells/mL) compared to the low colonized stomach or duodenum (10^2−4^ cells/mL) [4]. Oral cavity is a rather heavily colonised site, with saliva containing up to 10^9^ commensal bacterial per millilitre [5].

The oral microbiome has been shaped by the environment though the human evolution. Thus, changes in the diet, including the increased use of processed foods and refined sugar, the use of prebiotics, probiotics and synbiotics; expansion of antimicrobial therapies (e.g. antibiotics), or hygiene habits, smoking or alcohol consumption having influenced the composition of the microbiota in the oral cavity [6]. In fact, modern-day habits have been associated with the increase of diseases such as dental caries or periodontal disease (e.g. [7], [8], [9]).

The mouth is a heterogeneous environment that offers different habitats and nutrients that can support microbial communities (teeth, gums, tongue, palate, etc.). More than 700 different prokaryote taxa have been identified in the mouth [10], with one third of them known only as uncultivated phylotypes.

Bacteria in the oral microbiota are organised forming complex aggregates called biofilms consisting of numerous species, which create collaborations and antagonisms, and are critical to either maintain oral health though their metabolic, immune and colonisation resistance functions or revert to disease by perturbation in their composition and/or functions [11].

Despite viruses being the most abundant infectious agents in different habitats, including the human body, relatively few studies have described viral communities in human oral cavity [12], [13], [14], compared to those focused on their bacterial counterparts. Those viruses are predominantly bacteriophages and differed from viromes described for other habitats. Counts of virus-like particles (VLP) based on epifluorescence microscopy have shown approximately 10^8^ VLP per millilitre of fluid from saliva and oropharyngeal swabs [13], [15], and 10^7^ of them per milligram of dental plaque [16]. Finding of virulence factor homologs in oral viromes suggests that viruses may serve as reservoirs for pathogenic gene functions, and also may have an important role in shaping the oral bacterial ecology in the oral environment [17].

In this work, we have analysed oral wash samples from a randomized population of healthy young adults in Valencia (Spain) for a metagenomic analysis through the construction of shotgun libraries and subsequent high-throughput-sequencing with the aim of characterising taxonomically the oral viral communities as well as and the gene content the latter from the oral cavity of this population.

## Materials and methods

### Ethic statement

This study was approved by the Ethics Committee for Clinical Research of the Directorate General of Public Health and Centre for Advanced Research in Public Health (CEIC DGSP / CSISP) to Dr Javier Diez. The study involved human participants. Oral consents were given and all data were analyzed anonymously. In order to be included in the study, volunteers were informed and provided informed consent.

### Subjects

Undergraduate students of 18 to 25 years of age attending the University of Valencia during the academic year 2012–2013 were asked to participate in this transversal study. Recruitment was performed in November 2012. Participants were kept anonymous and only the variable sex was recorded.

### Sample collection

Each participant was provided with a tube containing 15 mL of sterile saline serum (NaCl 0.9%). Samples were collected after thorough oral rinse of the oral cavity for 30 seconds. Fresh specimens were kept refrigerated during transport and then frozen at –20°C at the laboratory facilities. For the nucleic acids extraction, samples were thawed on ice, and centrifuged at 4,000 x g for 5 min, at 4°C. Supernatants were transferred into fresh sterile 15 mL tubes. Aliquots were distributed among sterile 2 mL tubes and dried out by speed-vac. Finally, all pellets from each sample were resuspended using 2 mL of Hanks Balanced Salt Solution (HBSS, GIBCO Gaithersburg, MD, USA) at 4°C and put together in a single 2 mL tube. The concentrated suspension was next filtered through a 0.22 μm pore size Acrodisc® Syringe filters (Pall España, Madrid, Spain) and the collected volume was treated with 12.5 units of 25U/μl Benzonase (Novagen Inc., Madison, WI, USA) for 120 min at 37°C to remove unprotected nucleic acids of bacterial or eukaryotic origin. Next, SDS and proteinase K were added to final concentrations of 1% and 0.05 mg/mL, respectively. After incubation at 55°C for 60 min, viral nucleic acids extractions were carried out by addition of 1V of phenol:chloroform:isoamyl alcohol 25:24:1, followed by a 24:1 mixture of chloroform-isoamyl alcohol. Precipitated nucleic acids were resuspended and quantified using a Qubit^®^ 3.0 Fluorometer (Thermo Fisher Scientific, Carlsbad, CA, USA). Shotgun libraries were prepared using the Nextera XT DNA Library Prep kit (Illumina, CA, USA). Sequencing was carried out by MiSeq Paired End Illumina Technology, using the Reagent Kit V3 for 300 bp paired-end reads. An additional aliquot of sterile saline serum was extracted in parallel to the samples for sequencing, as control of contamination.

### Sequence pre-processing

Bioinformatic pre-processing of the viral metagenomes was performed as follows. FASTQ-format files containing raw sequence reads, were quality and length- filtered using Prinseq-lite (v0.20.4) [18], which also implemented the removal of reads containing polynucleotide tails, Ns and low complexity sequences. Next, trimmed reads showing average quality below 20 were concordantly removed. The forward and reverse reads were concordantly joined with Fastq Joiner [19], and unjoined reads remained in separate files from the joined ones.

### Taxonomic analyses

Files were converted into FASTA format. Subsequently, host reads were filtered using Bowtie2 [20], with end-to-end and very sensitive options for mapping against the GRCh37 reference human genome. The resulting files were the input for a mapping against a bacterial database (Bacteria_2016_02_12), based on the reference bacterial genomes updated to February 2016. the unmapped reads were used for a BLASTn search (e-value <10^−3^, identity ≥ 80% along ≥ 75% of the read length) against a viral genomes database (VirusDB_2016_03_21). This database consisted of 99% identity clusters of a collection of complete viral genomes from the EBI and NCBI sites, plus all available viral sequences from the International Nucleotide Sequence Database Collaboration (INSDC), updated on March 2016, as well as prophages from PHAge Search Tool (PHAST) [21]. Thus, potential viral hits were identified when present in our database. In both cases, an in-house script was then used to compare the identity and alignment length of the different potential hits matching each query and a lowest common ancestor (LCA) strategy was used to assign the best hit when possible to the closest-to-the–tip taxonomic level, or an undetermined hit if not possible.

Finally, the resulting single hits per query for each sample were used to construct operational taxonomic unit (OTU) tables that were converted into BIOM format for the analysis with QIIME version 1.8.0 [22]. Sample composition, abundance and diversity within samples (as well as within groups of samples) were calculated. In order to compare groups of samples, for example females vs. males, the same number of reads per sample were subsampled, using two different conditions, less and more stringent, so that more and fewer samples passed the filters respectively.

The alpha diversity was calculated with Shannon diversity index, implemented by QIIME pipeline. Boxplots were created and the diversity compared by groups using the script *compare_alpha_diversity*.*py* and using a non-parametric two-tailed t-test using Monte Carlo permutations with FDR correction [22].

The beta diversity was calculated using the Bray-Curtis dissimilarity index, and distances within and between females and males were assessed using the script *make_distance_boxplots*.*py*, carrying out Monte Carlo (nonparametric) tests, including Bonferroni corrections [22].

### Data submission

The DNA virus metagenome data sets from this study are available in the EBI Short Read Archive under the study accession number PRJEB22882, with accession numbers [ERS1960382—ERS1960453].

## Results

The viral metagenomes of 72 healthy young Spanish people were taxonomically analysed, and also the potential gene content was studied in the case of bacteria. A summary of the samples ID and gender of the subjects participating in the study is displayed in S1 Table.

Sequencing results can be seen at S2 Table in SI. The sequencing of those samples resulted in 27,686,743 pairs of reads, which after bioinformatics processing became 1,339,784 bacterial reads, and 204,057 viral reads (184,078 of them showing also homology to bacteria, and 19,979 reads homologous to the viral but not to bacterial database).

A more detailed view into the viral hits reveals that hits against prophages accounted for 92.0% of the reads compared to the 8.0% of bacteriophages and eukaryotic viruses when all reads are considered. However, both types of sequences were more evenly distributed (51.7% vs. 48.3%, respectively) if only viral reads with no homology to bacterial databases were considered. Contrarily, if only viral reads showing also homology to bacterial databases were considered, the bias towards prophages increased to 96.3% compared to only 3,7% of viral hits.

### Sample range distribution

**Fig 1:**
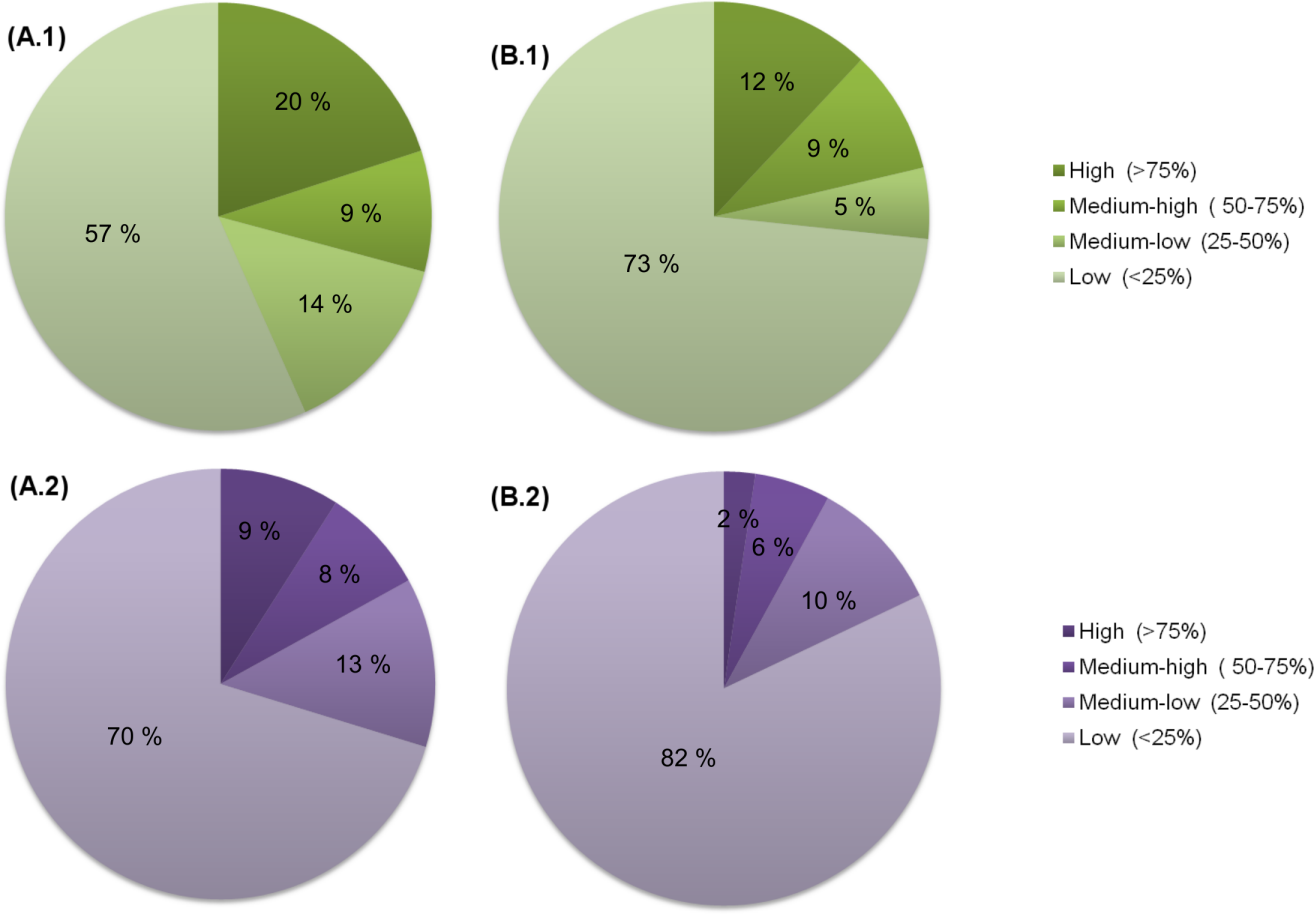
Pie charts showing the relative distribution of the viral reads through the samples, based on their presence/absence. According to the percentage of samples where a particular viral hit is identified, four categories are shown (high, medium-high, medium-low and low). Composition for reads with homology to viruses and bacteria is displayed at family (A.1) and species level (A.2). Composition for reads with homology to viruses but without homology to bacteria is also displayed at family (B.1) and species level (B.2). Green spectrum indicates family taxonomic level and purple spectrum indicates species level.

Viral reads composition was analysed across samples by reporting their presence/absence at the family and species taxonomic levels. According to the percentage of samples where a particular viral hit could be identified, viruses fell into four categories (Fig 1). Viral hits identified in more than 75% of the samples can be considered as ‘core’ viruses (i.e. shared by most of the population of study); those ranging from 50 to 75% and from 25 to 50% of the samples were frequently to moderately shared among the community, respectively, and those found in less than 25% of samples can be considered as individual-specific hits. If all hits were considered (Fig 1A.1 and 1A.2), the distribution pattern was always that of a majority of viruses found in few individuals (57% and 70% at family and species level, respectively), with single occurrences being the most widespread category (19.2% and 30.6% of all viral families and species, respectively). On the contrary, universally distributed viruses were relatively rare, with 20% and 9% of the viral families and species falling within this category. In addition to a series of prophages matching those identified in the genome of bacteria belonging to families Fusobacteriaceae, Streptococcaceae, Neisseriaceae and Veillonellaceae (all of them identified in 71 samples), the most widespread viruses included two families of bacteriophages within the order *Caudovirales*, *Siphoviridae* (71 samples) and *Myoviridae* (68 samples), as well as several *Streptococcus* phages (up to 69 samples). The most widespread eukaryotic viruses belonged to the *Herpesviridae* family (65 samples), in particular *Human herpesvirus 7*, identified in 61 out of the 72 samples.

If reads matching also bacterial sequences are excluded from the analysis, thus considering strictly those reads matching only viruses were considered (Fig 1B.1 and 1B.2), bacteriophages from family *Siphoviridae* (69), *Myoviridae* (56) and *Streptococcus* phages (59), as well as the *Herpesviridae* (63) were still falling amongst the most widespread viruses. As for prophages, fewer are described in this category, but still those from Streptococcaceae (70), Neisseriaceae (63) and Veillonellaceae (59) bacteria were highly widespread, while those from Fusobacteriaceae (43) fell more sharply. Those results indicate that, despite the differences observed between considering all hits or only those matching only viruses, the main observations remain essentially unchanged, supporting the existence of a consistent core of viruses and prophages regardless of the inclusion/exclusion of mixed bacterial and viral hits.

The complete list of prophages, bacteriophages and eukaryotic viruses as well as the number of occurrences in samples are displayed in S3 Table and S1 Fig in SI, respectively.

**Fig 2:**
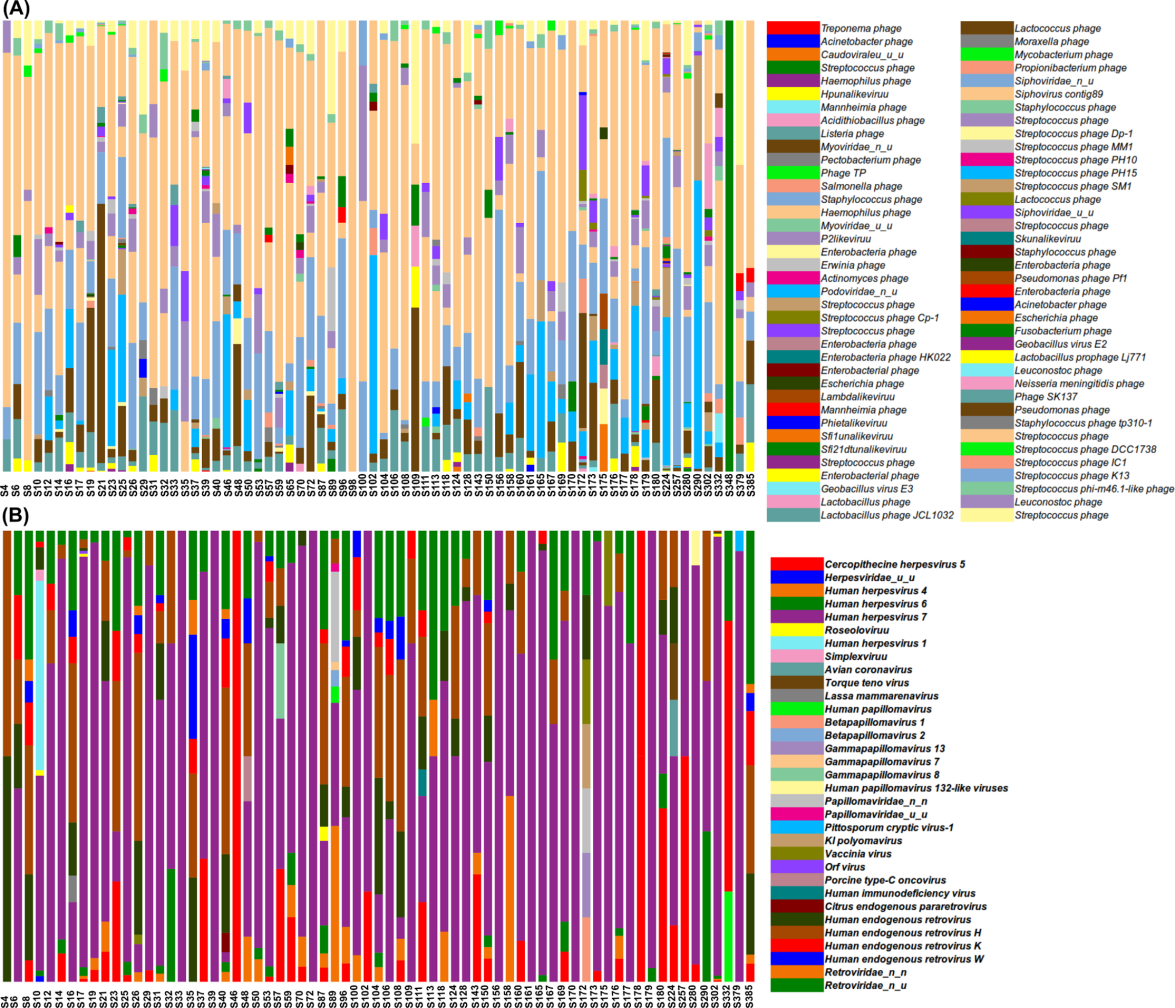
**Barplots showing the relative abundance of the bacteriophages (A) and eukaryotic viruses (B), at the species level, identified by sample.** The order of the viruses in the legend is based on the alphabetical order of their full taxonomic assignment (see S4 Table in SI for it). Viruses displayed in the legend are sorted in ascending order in the bars, i.e., the first viruses in the legend appear at the bottom of the bar, whereas the last ones are displayed at the top of the bars.

In addition to the composition analyses, based on the detection of presence/absence of a particular viral hit in the samples, viral hits were counted allowing us to estimate the absolute and relative abundance of the different viral taxa across samples. Fig 2 shows the relative abundance of bacteriophages (A) and eukaryotic viruses (B) in the individual samples. Several *Streptococcus* phages (26.1, 4.4 and 2.3%), *Siphovirus contig89* (12.3%), *Listeria phage* (2.2%), and undetermined members of *Siphoviridae*, *Podoviridae* and *Myoviridae* families accounted for more reads in the category of bacteriophages. The most abundant reads among eukaryotic viruses belonged to herpesviruses (74%), in particular *Human herpesvirus* (66%), followed by retroviruses (24%), and papillomaviruses (1.2%), with only occasional counts of poxviruses, conronaviruses, anelloviruses, and others (less than 1% of all reads).

A more detailed list of the bacteriophages and eukaryotic viruses identified in each sample, including their full taxonomic assignment, and their absolute counts and relative abundance, is displayed in S4 Table.

### Viral diversity

**Fig 3:**
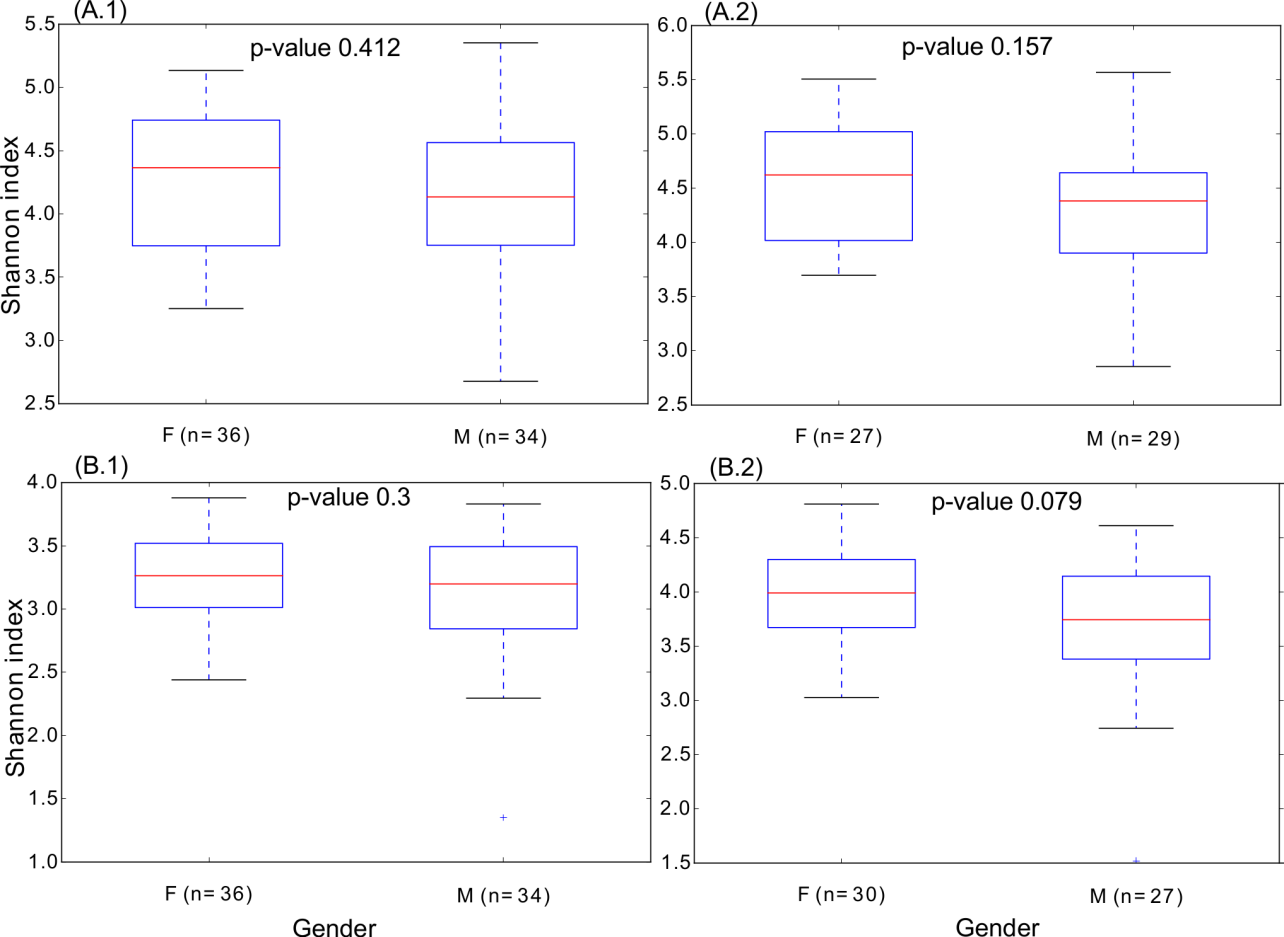
**Boxplots showing the alpha diversity of samples according to the sex, for viruses (A, B).** The diversity within samples, calculated for the groups of females (F) and males (M) samples, is displayed for read matching simultaneously viral and bacterial databases (A.1 and A.2), and for reads matching virus database solely (B.1, B.2). The number of reads subsampled was, in the case of viruses, approached in a less strict approach, (A.1, B.1) using also a stricter approach with an increased number of reads subsampled (A.2, B.2). Those figures were 157 (A.1), 700 (A.2), 22 (B.1) and 80 (B.2).

Alpha diversity, measured by the Shannon diversity index, is shown in Fig 3. For all viruses, 70 and 56 samples passed the threshold of subsampling 157 and 700 reads per sample, respectively (Fig 3A.1 and 3A.2). For virus-only hits, subsampling 22 and 80 reads allowed obtaining 70 and 57 samples above those thresholds, respectively (Fig 3B.1 and 3B.2). Regardless of the inclusion of virus-only hits or virus plus bacterial hits, and also the number or reads used for subsampling for comparable results, differences by gender were not significant, (p-value >0.05) and the patterns were similar in all cases.

As for beta diversity, comparison of distances using the dissimilarity Bray-Curtis index also revealed no significant differences according to the sex in any of the comparisons (S2 Fig in SI), for viral hits also matching bacteria, (A.1) as well as for only-virus hits (A.2).

## Discussion

The oral cavity is a heterogeneous environment, with factors such as diet, prebiotics and probiotics, drugs, hygiene habits, smoking or alcohol consumption influencing the composition of the microbiota, and therefore, changes linked to modern-life habits, may have an impact on it. The study of the oral cavity virome has been already the focus of different research analyses during the last years, focused mostly on unstimulated saliva, dental plaque or oral swabs from healthy individuals (e.g. [13], [23], [16], [24], [25]), but also on diseases, such as periodontal disease [14] or other disturbances, such as antibiotic treatment [26], with extensive reviews [17], [27]. Normally, sample size has been an issue, with small numbers of subject in the studies, ranging from four individuals [16] to 21 [23], and only a deep survey achieved samples from 102 subjects [25]. In this study, we report the results of the analysis based primarily on the DNA virome from the oral cavity from 72 healthy individuals, with a balanced ratio by sex and using oral wash as the source of samples. We have also included taxonomic and functional results from the oral bacterial metagenome from the same set of samples, in order to provide a wider view of the oral microbiome form the analysed population. Despite the relatively low depth of the sequencing, which may hinder some analyses, our samples provide a global and unspecific overview of the DNA viral communities with similarity to those form extant databases inhabiting the oral cavity using a simple and non-invasive sample collection, that allows for an extensive population study.

The idea of a stable core virome has already been pointed out for the respiratory tract [28], the gut [29], or the skin [30], although in the last case, a distinction is made between eukaryotic viruses and bacteriophages, with little evidence of a core for the former but the presence of a strong core for the latter. Our results support, for the case of the oral virome, the existence of a small proportion of prophages, bacteriophages and eukaryotic viruses that are found in most of the samples analysed, such as prophages and bacteriophages from several *Streptococcus* species, as well as *Human herpesvirus 7*. Interestingly, the latter was already found to be widely distributed across samples in the study by [25], under the genus *Roseolovirus*, with a presence > 90% of their individuals, suggesting that this group of herpesvirus is universally found regardless of the human population studied, at least for Western society. Also concordantly to their results, our findings of papillomaviruses are limited to the identification of few alpha- beta and gamma-papillomaviruses and in five samples, suggesting that, unlike other tissues, like nose, skin or vagina, these viruses are not as prevalent.

At any rate, highly prevalent viruses or core viruses are largely outnumbered by viruses that are found sporadically in a single or few samples. As expected, at higher taxonomic level, the aggregation of several species within a family, produces a noticeably increase in the core virome relative to the species level.

In a study on eight subjects, differences by sex in the oral virome were reported [24] in terms of viral homologs. However, in our study on 72 subjects we have not found significant differences in the diversity of viruses and bacteria between females and males in any comparison.

Since our main goal was to describe the viral community form the oral cavity in a specific population of Spanish young adults from 18–25 years old, our study is cross-sectional and only includes samples collected at a single time point per individual, and thus we cannot assess the temporal variability of either the viruses or bacteria, but the relatively large sample size allows us to envisage some traits of the oral microbiome that appear to be rather universal when it comes to the oral viruses, regardless of the geographical area.

Future perspectives include a deeper sequencing of these and other samples, together with the inclusion of the analysis of RNA viruses in order to provide a better picture of the viral communities, by obtaining a significant number of reads that would allow us to explore in more detail, in the non-human, non-bacterial universe, the ‘viral dark matter’ [31] from the remaining unidentified reads, in search of novel putative viruses.

## Supporting information

S1 Table Samples ID and gender of the subjects participating in the study. (DOC)

S2 Table Summary of the number of reads per samples during the different steps of bioinformatics processing. The resulting number reads after each filtering step (i.e. quality and length filtering, low-complexity & Ns filtering, joining, and removal of human-origin reads) are shown for each samples as well as globally.
(XLSX)

S3 Table List of prophages, bacteriophages and eukaryotic DNA viruses, and distribution by range of occurrences through the samples of the study. According to their relative presence, four categories are shown (high, medium-high, medium-low and low), plus an additional column displaying the cases of occurrence in only one sample (singletons). Figures in red indicate the number cases of viral hits identified within each category as well as the total number of occurrences. Hits are sorted, within each category, in descending order of occurrences. Comparisons are displayed for reads with homology to viruses and bacteria, at family level (A); with homology to viruses and bacteria, at species level (B); reads with homology to viruses but without homology to bacteria at family level (C); and reads with homology to viruses but without homology to bacteria at species level (D). Black bold letters indicate bacteriophages, dark red bold letters indicate eukaryotic viruses, and the remaining cases indicate prophages.
(DOC)

S4 Table Table of absolute counts and relative abundance (expressed as a percentage) of the bacteriophages and eukaryotic viruses identified in the set of samples. Bacteriophages are indicated in regular italics, while eukaryotic viruses are indicated in bold italics. Full taxonomic assignments are displayed. Colours in the first column match those of Fig 2. o_: order, f_: family, g_: genus; s_: species; _u: undetermined; _n: no name in the database for that taxonomic rank.
(XLSX)

S1 Fig Histograms of abundance of occurrences of the different prophages, bacteriophages and eukaryotic viruses through the samples, at family and species level. Number of occurrences for reads with homology to viruses and bacteria is displayed at family (A.1) and species level (A.2); whereas for reads with homology to viruses but without homology to bacteria is displayed at family (B.1) and species level (B.2). The four categories described (high, medium-high, medium-low and low) are shown. Green spectrum indicates family taxonomic level and purple spectrum indicates species level.
(TIF)

S2 Fig Boxplots showing the overall beta diversity of the samples as well as the distribution by sex, based on Bray-Curtis dissimilarity. The maximum, 3^rd^ quartile, median, 1^st^ quartile and minimum values are depicted for each box and whisker. Outlier are also plotted. F: females; M males; FM: females-males. The statistical significance, based on Monte Carlo (nonparametric) tests with Bonferroni corrections, is also reported for the comparisons among sexes.
(TIF)
