# Supplementary material for: The analysis of the oral DNA virome reveals which viruses are widespread and rare among healthy young adults in Valencia (Spain)
Source: PLoS One. 2018 Feb 8;13(2):e0191867. doi: 10.1371/journal.pone.0191867 (PMC5805259; doi:10.1371/journal.pone.0191867)
Supplement: S1 Table — (DOC) [file pone.0191867.s001.doc]

**Table 1. Samples ID and gender of the subjects participating in the study.** Identification of human papillomaviruses (HPV) is also shown.

| **#Sample ID** | Gender | HPV screening |  | **#Sample ID** | Gender | HPV screening |
| --- | --- | --- | --- | --- | --- | --- |
| S004 | F | - |  | S104 | M | - |
| S006 | F | - |  | S106 | F | - |
| S008 | F | - |  | S108 | F | - |
| S010 | F | - |  | S109 | M | - |
| S012 | F | - |  | S111 | M | - |
| S014 | F | - |  | S113 | F | - |
| S016 | F | - |  | S118 | F | + |
| S017 | M | - |  | S124 | M | - |
| S019 | M | - |  | S128 | F | + |
| S021 | F | - |  | S143 | M | - |
| S023 | M | - |  | S150 | F | + |
| S025 | M | - |  | S156 | F | - |
| S026 | M | - |  | S158 | F | + |
| S029 | M | - |  | S160 | M | + |
| S031 | F | - |  | S161 | M | - |
| S032 | M | - |  | S165 | M | - |
| S033 | M | - |  | S167 | M | - |
| S035 | F | - |  | S169 | M | - |
| S037 | M | - |  | S170 | F | - |
| S039 | M | - |  | S172 | M | + |
| S040 | M | - |  | S173 | M | - |
| S046 | F | - |  | S175 | M | - |
| S048 | F | - |  | S176 | F | - |
| S050 | F | - |  | S177 | M | - |
| S053 | F | - |  | S178 | M | - |
| S057 | F | - |  | S179 | F | - |
| S059 | F | - |  | S180 | M | - |
| S065 | F | - |  | S224 | F | + |
| S070 | F | + |  | S257 | M | + |
| S072 | M | - |  | S280 | M | + |
| S087 | F | - |  | S290 | M | + |
| S089 | F | - |  | S302 | M | + |
| S096 | M | - |  | S332 | F | + |
| S098 | F | - |  | S348 | F | + |
| S100 | F | - |  | S379 | M | + |
| S102 | F | - |  | S385 | F | + |
