# Supplementary material for: The analysis of the oral DNA virome reveals which viruses are widespread and rare among healthy young adults in Valencia (Spain)
Source: PLoS One. 2018 Feb 8;13(2):e0191867. doi: 10.1371/journal.pone.0191867 (PMC5805259; doi:10.1371/journal.pone.0191867)
Supplement: S3 Table — According to their relative presence, four categories are shown (high, medium-high, medium-low and low), plus an additional column displaying the cases of occurrence in only one sample (singletons). Figures in red indicate the number cases of viral hits identified within each category as well as the total number of occurrences. Hits are sorted, within each category, in descending order of occurrences. Comparisons are displayed for reads with homology to viruses and bacteria, at family level (A); with homology to viruses and bacteria, at species level (B); reads with homology to viruses but without homology to bacteria at family level (C); and reads with homology to viruses but without homology to bacteria at species level (D). Black bold letters indicate bacteriophages, dark red bold letters indicate eukaryotic viruses, and the remaining cases indicate prophages. (DOC) [file pone.0191867.s003.doc]

(A)

| **Presence** | | | | |
| --- | --- | --- | --- | --- |
| **High (>75%)** | **Medium-high (50-75%)** | **Medium-low (25-50%)** | **Low (<25%)** | **Singletons** |
| **24** | **11** | **17** | **68** | **23** |
| **120** | | | |
| Fusobacteriaceae_phage | Corynebacteriaceae_phage | Rhodocyclaceae_phage | Leptotrichiaceae_phage | Acidimicrobiaceae_phage |
| Streptococcaceae_phage | Erysipelotrichaceae_phage | Atopobiaceae_phage | undetermined | Alteromonadaceae_phage |
| Neisseriaceae_phage | Pseudomonadaceae_phage | Bacillaceae_phage | Aerococcaceae_phage | Shewanellaceae_phage |
| Veillonellaceae_phage | Staphylococcaceae_phage | Clostridiales_n_phage | Clostridiaceae_phage | Paenibacillaceae_phage |
| undefined | **u_u_Streptococcus phage** | Spirochaetaceae_phage | Bacteroidaceae_phage | Eubacteriaceae_phage |
| **Siphoviridae** | **Podoviridae** | Bacillales_phage_u | Ruminococcaceae_phage | Desulfarculaceae_phage |
| Pasteurellaceae_phage | Lactobacillaceae_phage | Oxalobacteraceae_phage | Leuconostocaceae_phage | Legionellaceae_phage |
| Prevotellaceae_phage | Moraxellaceae_phage | **n_n_Fusobacterium phage** | Micrococcaceae_phage | Methylococcaceae_phage |
| **n_n_Streptococcus phage** | Actinomycetaceae_phage | Clostridiales_phage_u | Listeriaceae_phage | Hyphomicrobiaceae_phage |
| **Myoviridae** | Cardiobacteriaceae_phage | Xanthomonadaceae_phage | Lactobacillales_phage_n | Methylocystaceae_phage |
| Peptostreptococcaceae_phage | **n_n_Streptococcus phi-m46.1-like phage** | Bacteroidales_phage_u | **n_n_Phage SK137** | Phyllobacteriaceae_phage |
| Carnobacteriaceae_phage |  | Streptomycetaceae_phage | Alcaligenaceae_phage | Thermaceae_phage |
| **Herpesviridae** |  | Burkholderiaceae_phage | Mycobacteriaceae_phage | n_Polymorphum_phage |
| **undefined** |  | Comamonadaceae_phage | n_n_uncultured bacterium_phage | u_u_Escherichia coli_phage |
| Lachnospiraceae_phage |  | Flavobacteriaceae_phage | Vibrionaceae_phage | **Caudovirales_n** |
| Enterobacteriaceae_phage |  | **n_n_Streptococcus phage DCC1738** | **Caudovirales_u** | **Anelloviridae** |
| Lactobacillales_phage_u |  | **n_n_Streptococcus phage K13** | **n_n_Neisseria meningitidis phage** | **Polyomaviridae** |
| Bifidobacteriaceae_phage |  |  | Colwelliaceae_phage | **n_n_Escherichia phage** |
| Enterococcaceae_phage |  |  | Peptococcaceae_phage | **n_n_Geobacillus virus E2** |
| **Retroviridae** |  |  | Sphingomonadaceae_phage | **n_n_Lactobacillus prophage Lj771** |
| Porphyromonadaceae_phage |  |  | **n_n_Streptococcus phage IC1** | **n_n_Leuconostoc phage** |
| Bacillales_n_phage |  |  | Propionibacteriaceae_phage | **n_n_Staphylococcus phage tp310-1** |
| Campylobacteraceae_phage |  |  | Desulfovibrionaceae_phage | **u_u_Leuconostoc phage** |
| Peptoniphilaceae_phage |  |  | **Microviridae** |  |
|  |  |  | **Papillomaviridae** |  |
| |  | | --- | |  |  | Helicobacteraceae_phage |  |
|  |  |  | Pseudomonadales_phage_u |  |
|  |  |  | Rhodobacteraceae_phage |  |
|  |  |  | Acetobacteraceae_phage |  |
|  |  |  | **Poxviridae** |  |
|  |  |  | Aeromonadaceae_phage |  |
|  |  |  | Burkholderiales_phage_u |  |
|  |  |  | Ectothiorhodospiraceae_phage |  |
|  |  |  | Pseudonocardiaceae_phage |  |
|  |  |  | Bradyrhizobiaceae_phage |  |
|  |  |  | Acidaminococcaceae_phage |  |
|  |  |  | **Inoviridae** |  |
|  |  |  | Planococcaceae_phage |  |
|  |  |  | Brevibacteriaceae_phage |  |
|  |  |  | Nitrospiraceae_phage |  |
|  |  |  | Brucellaceae_phage |  |
|  |  |  | Rhizobiaceae_phage |  |
|  |  |  | Rhodospirillaceae_phage |  |
|  |  |  | **n_n_Acinetobacter phage** |  |
|  |  |  | **n_n_Pseudomonas phage** |  |
|  |  |  | Acidimicrobiaceae_phage |  |
|  |  |  | Alteromonadaceae_phage |  |
|  |  |  | Shewanellaceae_phage |  |
|  |  |  | Paenibacillaceae_phage |  |
|  |  |  | Eubacteriaceae_phage |  |
|  |  |  | Desulfarculaceae_phage |  |
|  |  |  | Legionellaceae_phage |  |
|  |  |  | Methylococcaceae_phage |  |
|  |  |  | Hyphomicrobiaceae_phage |  |
|  |  |  | Methylocystaceae_phage |  |
|  |  |  | Phyllobacteriaceae_phage |  |
|  |  |  | Thermaceae_phage |  |
|  |  |  | n_Polymorphum_phage |  |
|  |  |  | u_u_Escherichia coli_phage |  |
|  |  |  | **Caudovirales_n** |  |
|  |  |  | **Anelloviridae** |  |
|  |  |  | **Polyomaviridae** |  |
|  |  |  | **n_n_Escherichia phage** |  |
|  |  |  | **n_n_Geobacillus virus E2** |  |
|  |  |  | **n_n_Lactobacillus prophage Lj771** |  |
|  |  |  | **n_n_Leuconostoc phage** |  |
|  |  |  | **n_n_Staphylococcus phage tp310-1** |  |
|  |  |  | **u_u_Leuconostoc phage** |  |

**(B)**

| **Presence** | | | | |
| --- | --- | --- | --- | --- |
| **High (>75%)** | **Medium-high (50-75%)** | **Medium-low (25-50%)** | **Low (<25%)** | **Singletons** |
| **51** | **45** | **72** | **397** | **173** |
| **565** | | | |
| *Streptococcus pneumoniae_phage* | *Fusobacterium_phage_u* | *Peptoniphilus duerdenii_phage* | *Bulleidia extructa_phage* | *Acidimicrobium ferrooxidans_phage* |
| *Streptococcus_phage_u* | *Streptococcaceae_phage_u_u* | *Lachnospiraceae bacterium oral taxon 500_phage* | *Capnocytophaga sp. oral taxon 338_phage* | *Arcanobacterium haemolyticum_phage* |
| *undefined* | *Streptococcus anginosus_phage* | *Gardnerella vaginalis_phage* | *Acinetobacter_phage_u* | *Aeromonas salmonicida_phage* |
| *Fusobacterium periodonticum_phage* | *Selenomonas sp. oral taxon 137_phage* | *Acinetobacter baumannii_phage* | *Lactobacillus iners_phage* | *Marinobacter hydrocarbonoclasticus_phage* |
| *Streptococcus infantis_phage* | *Streptococcus sp. I-P16_phage* | *Azospira oryzae_phage* | *Lactobacillus_phage_u* | *Shewanella baltica_phage* |
| *Streptococcus mitis_phage* | *Selenomonas ruminantium_phage* | *Dialister invisus_phage* | *Streptomyces scabiei_phage* | *Bacillus licheniformis_phage* |
| *Streptococcus oralis_phage* | *Bifidobacterium sp. 12_1_47BFAA_phage* | *Catonella morbi_phage* | *undetermined* | *Geobacillus sp. JF8_phage* |
| *Streptococcus sanguinis_phage* | *Neisseria macacae_phage* | *Lachnospiraceae_n_phage_u* | *Bacillaceae_phage_u_u* | *Geobacillus thermoglucosidasius_phage* |
| *Neisseria gonorrhoeae_phage* | *Actinobacillus pleuropneumoniae_phage* | *Pasteurella multocida_phage* | *Alicycliphilus denitrificans_phage* | *Geobacillus_phage_u* |
| *Haemophilus influenzae_phage* | *Campylobacter concisus_phage* | *Megasphaera sp. UPII 135-E_phage* | *Campylobacter gracilis_phage* | *Paenibacillus larvae_phage* |
| *Neisseria meningitidis_phage* | *Escherichia coli_phage* | ***Cercopithecine herpesvirus 5*** | *Abiotrophia defectiva_phage* | *Staphylococcus caprae_phage* |
| *Neisseria_phage_u* | *Streptococcus salivarius_phage* | ***Human endogenous retrovirus*** | *Treponema socranskii_phage* | *Staphylococcus haemolyticus_phage* |
| ***Streptococcus phage*** | *Streptococcus sp. oral taxon 056_phage* | *Lachnospiraceae_phage_u_u* | *Propionibacterium phage* | *Staphylococcus xylosus_phage* |
| *Streptococcus vestibularis_phage* | *Streptococcus sp. oral taxon 071_phage* | *Selenomonas_phage_u* | ***Human endogenous retrovirus W*** | *Bacteroides sp. 2_2_4_phage* |
| *Haemophilus_phage_u* | *Selenomonas sp. oral taxon 149_phage* | *Staphylococcus aureus_phage* | *Prevotella dentalis_phage* | *Achromobacter piechaudii_phage* |
| *Fusobacterium nucleatum_phage* | *Aggregatibacter sp. oral taxon 458_phage* | *Clostridiales bacterium 1_7_47FAA_phage* | *Corynebacterium matruchotii_phage* | *Achromobacter xylosoxidans_phage* |
| *Veillonella dispar_phage* | ***Streptococcus phage*** | *Neisseria sp. oral taxon 014_phage* | *Klebsiella pneumoniae_phage* | *Burkholderia multivorans_phage* |
| *Veillonella parvula_phage* | *Peptoclostridium difficile_phage* | *Haemophilus pittmaniae_phage* | *Streptococcus infantarius_phage* | *Burkholderia_phage_u* |
| *Prevotella marshii_phage* | *Corynebacterium jeikeium_phage* | *Centipeda periodontii_phage* | *Streptococcus intermedius_phage* | *Cupriavidus necator_phage* |
| *Prevotella veroralis_phage* | *Veillonella sp. 3_1_44_phage* | ***Siphoviridae_u_u*** | *Avibacterium paragallinarum_phage* | *Ralstonia pickettii_phage* |
| *Granulicatella adiacens_phage* | *Prevotella nigrescens_phage* | *Gemella morbillorum_phage* | *Pseudomonas_phage_u* | *Acidovorax citrulli_phage* |
| *Neisseria flavescens_phage* | *Enterococcus faecium_phage* | *Oribacterium sinus_phage* | *Anaerococcus hydrogenalis_phage* | *Delftia acidovorans_phage* |
| *Neisseria lactamica_phage* | *Streptococcus pyogenes_phage* | *Neisseria bacilliformis_phage* | *Lachnoclostridium_phage_u* | *Delftia sp. Cs1-4_phage* |
| ***undefined*** | *Aggregatibacter segnis_phage* | *Bacillales_phage_u_u_u* | *Enterobacter cloacae_phage* | *Verminephrobacter eiseniae_phage* |
| *Streptococcus suis_phage* | *Solobacterium moorei_phage* | *Streptococcus agalactiae_phage* | *Aggregatibacter actinomycetemcomitans_phage* | *Herbaspirillum seropedicae_phage* |
| ***Siphoviridae_n_u*** | *Veillonellaceae_phage_u_u* | *Peptoniphilaceae_phage_u_u* | *Histophilus somni_phage* | *Campylobacter lari_phage* |
| *[Haemophilus] parasuis_phage* | *Peptostreptococcus stomatis_phage* | *Gemella sanguinis_phage* | *Selenomonas artemidis_phage* | *Clostridium kluyveri_phage* |
| *Pasteurellaceae_phage_u_u* | *Enterococcus_phage_u* | *Prevotella sp. oral taxon 299_phage* | *Finegoldia magna_phage* | *Hungatella hathewayi_phage* |
| *Filifactor alocis_phage* | ***Human endogenous retrovirus H*** | *Simonsiella muelleri_phage* | *Sfi21dtunalikevirus_u* | *Eubacterium saphenum_phage* |
| *Megasphaera micronuciformis_phage* | *Haemophilus parainfluenzae_phage* | *Parvimonas micra_phage* | *[Clostridium] asparagiforme_phage* | *Blautia obeum_phage* |
| *Streptococcus dysgalactiae_phage* | ***Retroviridae_n_u*** | *Mobiluncus_phage_n* | *Micrococcus luteus_phage* | *Coprococcus eutactus_phage* |
| *Haemophilus haemolyticus_phage* | *Streptococcus parasanguinis_phage* | *Peptostreptococcaceae_phage_u_u* | *Actinobacillus_phage_u* | *[Clostridium] symbiosum_phage* |
| *Veillonella atypica_phage* | *Aggregatibacter_phage_u* | *Selenomonas noxia_phage* | *Acinetobacter lwoffii_phage* | *Lachnospiraceae bacterium 5_1_63FAA_phage* |
| ***Human herpesvirus 7*** | *Gemella haemolysans_phage* | *Fusobacterium phage* | ***Moraxella phage*** | *Lachnospiraceae bacterium 6_1_37FAA_phage* |
| *Lactobacillales_phage_u_u_u* | *Cardiobacterium hominis_phage* | *Clostridiales_phage_u_u_u* | ***Geobacillus sp. Y4.1MC1_phage*** | *Lachnospiraceae bacterium 6_1_63FAA_phage* |
| *Streptococcus constellatus_phage* | *Neisseria mucosa_phage* | *[Eubacterium] rectale_phage* | *Listeria innocua_phage* | *Roseburia intestinalis_phage* |
| ***Siphovirus contig89*** | *Mannheimia haemolytica_phage* | *Atopobium rimae_phage* | *[Ruminococcus] torques_phage* | *Desulfitobacterium hafniense_phage* |
| *Prevotella pallens_phage* | ***Podoviridae_n_u*** | *Streptococcus sp. C150_phage* | *Shigella dysenteriae_phage* | *Acetivibrio cellulolyticus_phage* |
| *Haemophilus aegyptius_phage* | *Streptococcus mutans_phage* | *Selenomonas sputigena_phage* | *Shigella flexneri_phage* | *Ethanoligenens harbinense_phage* |
| *Streptococcus pseudopneumoniae_phage* | *Kingella oralis_phage* | *Veillonella sp. 6_1_27_phage* | *Leptotrichia hofstadii_phage* | *Corynebacterium argentoratense_phage* |
| *Prevotella_phage_u* | *Prevotella bivia_phage* | ***Hpunalikevirus_u*** | *Lactobacillales_phage_n_n_n* | *Corynebacterium aurimucosum_phage* |
| *Enterobacteriaceae_phage_u_u* | *Enterococcus faecalis_phage* | ***Human endogenous retrovirus K*** | ***Phage SK137*** | *Corynebacterium pseudogenitalium_phage* |
| *Neisseria sicca_phage* | *Neisseria elongata_phage* | *Mobiluncus curtisii_phage* | *Mobiluncus mulieris_phage* | *Mycobacterium avium_phage* |
| ***Listeria phage*** | *Pseudomonas putida_phage* | *Bacteroidales_phage_u_u_u* | *Bacillus_phage_u* | *Mycobacterium kansasii_phage* |
| ***Myoviridae_n_u*** | *Streptococcus phi-m46.1-like phage* | *Bifidobacterium pseudocatenulatum_phage* | *Bifidobacterium bifidum_phage* | *Desulfarculus baarsii_phage* |
| *Neisseria polysaccharea_phage* |  | *Lachnoanaerobaculum saburreum_phage* | *Shuttleworthia satelles_phage* | *Desulfovibrio sp. 6_1_46AFAA_phage* |
| *Veillonella_phage_u* |  | *Treponema denticola_phage* | *Faecalibacterium prausnitzii_phage* | *Citrobacter koseri_phage* |
| *Barnesiella viscericola_phage* |  | ***Human herpesvirus 4*** | *Lactobacillus salivarius_phage* | *Citrobacter rodentium_phage* |
| *Aggregatibacter aphrophilus_phage* |  | *Pseudomonas aeruginosa_phage* | ***Streptococcus phage PH10*** | *Citrobacter youngae_phage* |
| ***Streptococcus phage*** |  | ***Streptococcus phage MM1*** | *Bacteroides thetaiotaomicron_phage* | *Edwardsiella piscicida_phage* |
| *Neisseria subflava_phage* |  | *Actinomyces viscosus_phage* | *Bifidobacterium_phage_u* | *Enterobacter asburiae_phage* |
| |  | | --- | |  | *Oxalobacter formigenes_phage* | *Lachnospiraceae bacterium 3_1_46FAA_phage* | *Enterobacter mori_phage* |
|  |  | *Campylobacter curvus_phage* | *Escherichia_phage_u* | *Enterobacter sp. 638_phage* |
|  |  | *Streptococcus gallolyticus_phage* | *Lactobacillus gasseri_phage* | *Enterobacter sp. R4-368_phage* |
|  |  | *Streptococcus thermophilus_phage* | *Streptococcus_phage_n* | *Plautia stali symbiont_phage* |
|  |  | *Eikenella corrodens_phage* | *Xanthomonadaceae_phage_u_u* | *Enterobacteriaceae_phage_n_n* |
|  |  | *Staphylococcus epidermidis_phage* | *uncultured bacterium_phage* | *Erwinia billingiae_phage* |
|  |  | *Atopobium parvulum_phage* | ***Enterobacteria phage*** | *Escherichia sp. TW09231_phage* |
|  |  | *Streptococcus macedonicus_phage* | ***Lactococcus phage*** | *Klebsiella oxytoca_phage* |
|  |  | *Kingella kingae_phage* | *Clostridium sp. BNL1100_phage* | *Klebsiella variicola_phage* |
|  |  | ***Streptococcus phage*** | *Oribacterium sp. oral taxon 078_phage* | *Morganella morganii_phage* |
|  |  | ***Human herpesvirus 6*** | *Fusobacterium gonidiaformans_phage* | *Proteus penneri_phage* |
|  |  | *Staphylococcus_phage_u* | *Streptomyces_phage_u* | *Rahnella sp. Y9602_phage* |
|  |  | *[Clostridium] saccharolyticum_phage* | *Peptoniphilus sp. oral taxon 375_phage* | *Raoultella ornithinolytica_phage* |
|  |  | *Lactococcus lactis_phage* | ***Myoviridae_u_u*** | *Lacinutrix sp. 5H-3-7-4_phage* |
|  |  | *Actinobacillus ureae_phage* | ***Neisseria meningitidis phage*** | *Fusobacterium necrophorum_phage* |
|  |  | *Salmonella enterica_phage* | *Colwellia psychrerythraea_phage* | *Leptotrichia goodfellowii_phage* |
|  |  | *Streptococcus equi_phage* | *Staphylococcus pseudintermedius_phage* | *Carnobacterium maltaromaticum_phage* |
|  |  | *Neisseriaceae_phage_u_u* | *Acidovorax avenae_phage* | *Lactobacillaceae_phage_u_u* |
|  |  | *Haemophilus sputorum_phage* | *Campylobacter rectus_phage* | *Lactobacillus amylovorus_phage* |
|  |  | ***Streptococcus phage DCC1738*** | *Mycobacterium smegmatis_phage* | *Lactobacillus antri_phage* |
|  |  | ***Streptococcus phage K13*** | *Lactobacillus fermentum_phage* | *Lactobacillus buchneri_phage* |
|  |  |  | *Streptococcus iniae_phage* | *Lactobacillus farciminis_phage* |
|  |  |  | *Actinobacillus suis_phage* | *Lactobacillus paracasei_phage* |
|  |  |  | *Streptomyces venezuelae_phage* | *Lactobacillus plantarum_phage* |
|  |  |  | *Stenotrophomonas maltophilia_phage* | *Enterococcus italicus_phage* |
|  |  |  | *Xanthomonas fuscans_phage* | *Lactococcus_phage_u* |
|  |  |  | ***Haemophilus phage*** | *Streptococcus ictaluri_phage* |
|  |  |  | ***Staphylococcus phage*** | *Streptococcus parauberis_phage* |
|  |  |  | ***Lactococcus phage*** | *Streptococcus porcinus_phage* |
|  |  |  | ***Streptococcus phage IC1*** | *Legionella pneumophila_phage* |
|  |  |  | *Prevotella sp. oral taxon 472_phage* | *Methylococcus capsulatus_phage* |
|  |  |  | *Bifidobacterium longum_phage* | *Gallibacterium anatis_phage* |
|  |  |  | *Clostridium bornimense_phage* | *Acinetobacter johnsonii_phage* |
|  |  |  | *Desulfotomaculum nigrificans_phage* | *Acinetobacter nosocomialis_phage* |
|  |  |  | *Enterobacter hormaechei_phage* | *Acinetobacter pittii_phage* |
|  |  |  | *Leptotrichia buccalis_phage* | *Acinetobacter radioresistens_phage* |
|  |  |  | *Lactobacillus helveticus_phage* | *Pseudomonas chlororaphis_phage* |
|  |  |  | *Lactobacillus hilgardii_phage* | *Pseudomonas coronafaciens_phage* |
|  |  |  | *Leuconostoc mesenteroides_phage* | *Pseudomonas mendocina_phage* |
|  |  |  | *Kingella denitrificans_phage* | *Pseudomonas parafulva_phage* |
|  |  |  | *Neisseria_phage_n* | *Pseudomonas savastanoi_phage* |
|  |  |  | *Propionibacterium acnes_phage* | *Pseudomonas sp. M1_phage* |
|  |  |  | *Pseudomonas pseudoalcaligenes_phage* | *Pseudomonas sp. TJI-51_phage* |
|  |  |  | *Vibrio cholerae_phage* | *Pseudomonas syringae group genomosp. 3_phage* |
|  |  |  | ***Retroviridae_n_n*** | *Acinetobacter baumannii_phage* |
|  |  |  | *Bacillus subtilis_phage* | *Nitrobacter sp. Nb-311A_phage* |
|  |  |  | *Staphylococcus capitis_phage* | *Nitrobacter winogradskyi_phage* |
|  |  |  | *Staphylococcus pasteuri_phage* | *Rhodopseudomonas palustris_phage* |
|  |  |  | *Burkholderia pseudomallei_phage* | *Hyphomicrobium denitrificans_phage* |
|  |  |  | *Burkholderia sp. Ch1-1_phage* | *Methylocystis sp. ATCC 49242_phage* |
|  |  |  | *Ralstonia sp. 5_7_47FAA_phage* | *Mesorhizobium sp. LNJC372A00_phage* |
|  |  |  | *Janthinobacterium agaricidamnosum_phage* | *Agrobacterium tumefaciens_phage* |
|  |  |  | *Lachnospiraceae bacterium 1_1_57FAA_phage* | *Rhizobium sp. IRBG74_phage* |
|  |  |  | *Salmonella_phage_u* | *Paracoccus denitrificans_phage* |
|  |  |  | *Yersinia pestis_phage* | *Roseovarius sp. TM1035_phage* |
|  |  |  | *Fusobacterium varium_phage* | *Acidaminococcus_phage_u* |
|  |  |  | *Lactobacillus reuteri_phage* | *Novosphingobium pentaromativorans_phage* |
|  |  |  | *Oenococcus oeni_phage* | *Sphingobium yanoikuyae_phage* |
|  |  |  | *Lactococcus garvieae_phage* | *Sphingopyxis alaskensis_phage* |
|  |  |  | *Actinobacillus minor_phage* | *Sphingopyxis fribergensis_phage* |
|  |  |  | *Pseudomonas fluorescens_phage* | *Streptomyces collinus_phage* |
|  |  |  | *Pseudomonas stutzeri_phage* | *Streptomyces fulvissimus_phage* |
|  |  |  | *Anaerococcus tetradius_phage* | *Streptomyces griseoaurantiacus_phage* |
|  |  |  | *Xanthomonas campestris_phage* | *Thermus parvatiensis_phage* |
|  |  |  | ***Staphylococcus phage*** | *Anaerococcus lactolyticus_phage* |
|  |  |  | ***Enterobacteria phage*** | *Anaerococcus_phage_u* |
|  |  |  | *Bacillus sp. BT1B_CT2_phage* | *Peptoniphilus lacrimalis_phage* |
|  |  |  | *Burkholderia sp. KJ006_phage* | *Peptoniphilus sp. oral taxon 836_phage* |
|  |  |  | *Helicobacter pylori_phage* | *Peptoniphilus_phage_u* |
|  |  |  | *Desulfovibrio alaskensis_phage* | *Vibrio alginolyticus_phage* |
|  |  |  | *Cronobacter sakazakii_phage* | *Vibrio ichthyoenteri_phage* |
|  |  |  | *Shigella sp. SF-2015_phage* | *Stenotrophomonas sp. SKA14_phage* |
|  |  |  | *Shigella_phage_u* | *Xanthomonas axonopodis_phage* |
|  |  |  | *Enterococcus italicus_phage* | *Xanthomonas citri_phage* |
|  |  |  | *Lactobacillus casei_phage* | *Xylella fastidiosa_phage* |
|  |  |  | *Lactobacillus crispatus_phage* | *Polymorphum gilvum_phage* |
|  |  |  | *Lactobacillus johnsonii_phage* | *Escherichia coli_phage* |
|  |  |  | *Streptococcus pseudoporcinus_phage* | ***Treponema phage*** |
|  |  |  | *Neisseria weaveri_phage* | ***Acinetobacter phage*** |
|  |  |  | *Pseudomonas monteilii_phage* | ***Mannheimia phage*** |
|  |  |  | *Methyloversatilis universalis_phage* | ***Pectobacterium phage*** |
|  |  |  | *Xanthomonas_phage_u* | ***Phage TP*** |
|  |  |  | ***Caudovirales_u_u_u*** | ***Salmonella phage*** |
|  |  |  | ***Acidithiobacillus phage*** | ***Haemophilus phage*** |
|  |  |  | ***Streptococcus phage SM1*** | ***P2likevirus_u*** |
|  |  |  | ***Roseolovirus_u*** | ***Streptococcus phage Cp-1*** |
|  |  |  | *Bacillus thuringiensis_phage* | ***Enterobacteria phage HK022*** |
|  |  |  | *Macrococcus caseolyticus_phage* | ***Enterobacterial phage*** |
|  |  |  | *Bacteroides sp. 4_3_47FAA_phage* | ***Escherichia phage*** |
|  |  |  | *Prevotella disiens_phage* | ***Lambdalikevirus_u*** |
|  |  |  | *Bifidobacterium breve_phage* | ***Mannheimia phage*** |
|  |  |  | *Bordetella parapertussis_phage* | ***Streptococcus phage*** |
|  |  |  | *Bordetella_phage_u* | ***Enterobacterial phage*** |
|  |  |  | *Burkholderia vietnamiensis_phage* | ***Geobacillus virus E3*** |
|  |  |  | *Ralstonia solanacearum_phage* | ***Lactobacillus phage JCL1032*** |
|  |  |  | *Burkholderiales_phage_u_u_u* | ***Mycobacterium phage*** |
|  |  |  | *Comamonas testosteroni_phage* | ***Streptococcus phage PH15*** |
|  |  |  | *Ectothiorhodospira sp. PHS-1_phage* | ***Streptococcus phage*** |
|  |  |  | *[Bacteroides] pectinophilus_phage* | ***Skunalikevirus_u*** |
|  |  |  | *Enterobacter aerogenes_phage* | ***Herpesviridae_u_u*** |
|  |  |  | *Enterobacter_phage_u* | ***Human herpesvirus 1*** |
|  |  |  | *Klebsiella sp. MS 92-3_phage* | ***Simplexvirus_u*** |
|  |  |  | *Klebsiella_phage_u* | ***Torque teno virus*** |
|  |  |  | *Pectobacterium atrosepticum_phage* | ***Pseudomonas phage Pf1*** |
|  |  |  | *Salmonella bongori_phage* | ***Betapapillomavirus 1*** |
|  |  |  | *Lactobacillus suebicus_phage* | ***Betapapillomavirus 2*** |
|  |  |  | *Acinetobacter calcoaceticus_phage* | ***Gammapapillomavirus 13*** |
|  |  |  | *Acinetobacter sp. ATCC 27244_phage* | ***Gammapapillomavirus 7*** |
|  |  |  | *Pseudomonadales_phage_u_u_u* | ***Gammapapillomavirus 8*** |
|  |  |  | *Saccharopolyspora erythraea_phage* | ***Human papillomavirus 132-like viruses*** |
|  |  |  | *Ketogulonicigenium vulgare_phage* | ***Papillomaviridae_u_u*** |
|  |  |  | *Komagataeibacter medellinensis_phage* | ***KI polyomavirus*** |
|  |  |  | *Acidaminococcus intestini_phage* | ***Orf virus*** |
|  |  |  | *Sphingomonas sp. MM-1_phage* | ***Porcine type-C oncovirus*** |
|  |  |  | *Streptomyces_phage_n* | ***Human immunodeficiency virus*** |
|  |  |  | *Xanthomonas albilineans_phage* | ***Citrus endogenous pararetrovirus*** |
|  |  |  | ***Streptococcus phage*** | ***Escherichia phage*** |
|  |  |  | ***Staphylococcus phage*** | ***Geobacillus virus E2*** |
|  |  |  | ***Erwinia phage*** | ***Lactobacillus prophage Lj771*** |
|  |  |  | ***Actinomyces phage*** | ***Leuconostoc phage*** |
|  |  |  | ***Streptococcus phage*** | ***Staphylococcus phage tp310-1*** |
|  |  |  | ***Enterobacteria phage*** | ***Leuconostoc phage*** |
|  |  |  | ***Phietalikevirus_u*** |  |
|  |  |  | ***Sfi1unalikevirus_u*** |  |
|  |  |  | ***Lactobacillus phage*** |  |
|  |  |  | ***Enterobacteria phage*** |  |
|  |  |  | ***Vaccinia virus*** |  |
|  |  |  | *Mobiluncus_phage_u* |  |
|  |  |  | *Aeromonas hydrophila_phage* |  |
|  |  |  | *Bacillus cereus_phage* |  |
|  |  |  | *Sporosarcina newyorkensis_phage* |  |
|  |  |  | *Staphylococcus hominis_phage* |  |
|  |  |  | *Staphylococcus saprophyticus_phage* |  |
|  |  |  | *Staphylococcus warneri_phage* |  |
|  |  |  | *Bordetella pertussis_phage* |  |
|  |  |  | *Bordetella petrii_phage* |  |
|  |  |  | *[Clostridium] scindens_phage* |  |
|  |  |  | *Peptostreptococcus anaerobius_phage* |  |
|  |  |  | *[Clostridium] leptum_phage* |  |
|  |  |  | *Corynebacterium diphtheriae_phage* |  |
|  |  |  | *Corynebacterium_phage_u* |  |
|  |  |  | *Mycobacterium gilvum_phage* |  |
|  |  |  | *Citrobacter freundii_phage* |  |
|  |  |  | *Enterobacter cancerogenus_phage* |  |
|  |  |  | *Escherichia albertii_phage* |  |
|  |  |  | *Escherichia sp. TW15838_phage* |  |
|  |  |  | *Escherichia_phage_n* |  |
|  |  |  | *Pectobacterium carotovorum_phage* |  |
|  |  |  | *Serratia marcescens_phage* |  |
|  |  |  | *Shigella boydii_phage* |  |
|  |  |  | *Shigella sonnei_phage* |  |
|  |  |  | *Yersinia enterocolitica_phage* |  |
|  |  |  | *Lactobacillus brevis_phage* |  |
|  |  |  | *Lactobacillus delbrueckii_phage* |  |
|  |  |  | *Lactobacillus rhamnosus_phage* |  |
|  |  |  | *Leuconostoc carnosum_phage* |  |
|  |  |  | *Streptococcus lutetiensis_phage* |  |
|  |  |  | *Streptococcus urinalis_phage* |  |
|  |  |  | *Brevibacterium linens_phage* |  |
|  |  |  | *Nitrospira defluvii_phage* |  |
|  |  |  | *Haemophilus_phage_n* |  |
|  |  |  | *Acinetobacter junii_phage* |  |
|  |  |  | *Acinetobacter sp. P8-3-8_phage* |  |
|  |  |  | *Ochrobactrum anthropi_phage* |  |
|  |  |  | *Gluconacetobacter diazotrophicus_phage* |  |
|  |  |  | *Azospirillum lipoferum_phage* |  |
|  |  |  | *Streptomyces avermitilis_phage* |  |
|  |  |  | *Xanthomonas oryzae_phage* |  |
|  |  |  | ***Streptococcus phage Dp-1*** |  |
|  |  |  | ***Human papillomavirus*** |  |
|  |  |  | ***Papillomaviridae_n_n*** |  |
|  |  |  | ***Acinetobacter phage*** |  |
|  |  |  | ***Pseudomonas phage*** |  |
|  |  |  | *Acidimicrobium ferrooxidans_phage* |  |
|  |  |  | *Arcanobacterium haemolyticum_phage* |  |
|  |  |  | *Aeromonas salmonicida_phage* |  |
|  |  |  | *Marinobacter hydrocarbonoclasticus_phage* |  |
|  |  |  | *Shewanella baltica_phage* |  |
|  |  |  | *Bacillus licheniformis_phage* |  |
|  |  |  | *Geobacillus sp. JF8_phage* |  |
|  |  |  | *Geobacillus thermoglucosidasius_phage* |  |
|  |  |  | *Geobacillus_phage_u* |  |
|  |  |  | *Paenibacillus larvae_phage* |  |
|  |  |  | *Staphylococcus caprae_phage* |  |
|  |  |  | *Staphylococcus haemolyticus_phage* |  |
|  |  |  | *Staphylococcus xylosus_phage* |  |
|  |  |  | *Bacteroides sp. 2_2_4_phage* |  |
|  |  |  | *Achromobacter piechaudii_phage* |  |
|  |  |  | *Achromobacter xylosoxidans_phage* |  |
|  |  |  | *Burkholderia multivorans_phage* |  |
|  |  |  | *Burkholderia_phage_u* |  |
|  |  |  | *Cupriavidus necator_phage* |  |
|  |  |  | *Ralstonia pickettii_phage* |  |
|  |  |  | *Acidovorax citrulli_phage* |  |
|  |  |  | *Delftia acidovorans_phage* |  |
|  |  |  | *Delftia sp. Cs1-4_phage* |  |
|  |  |  | *Verminephrobacter eiseniae_phage* |  |
|  |  |  | *Herbaspirillum seropedicae_phage* |  |
|  |  |  | *Campylobacter lari_phage* |  |
|  |  |  | *Clostridium kluyveri_phage* |  |
|  |  |  | *Hungatella hathewayi_phage* |  |
|  |  |  | *Eubacterium saphenum_phage* |  |
|  |  |  | *Blautia obeum_phage* |  |
|  |  |  | *Coprococcus eutactus_phage* |  |
|  |  |  | *[Clostridium] symbiosum_phage* |  |
|  |  |  | *Lachnospiraceae bacterium 5_1_63FAA_phage* |  |
|  |  |  | *Lachnospiraceae bacterium 6_1_37FAA_phage* |  |
|  |  |  | *Lachnospiraceae bacterium 6_1_63FAA_phage* |  |
|  |  |  | *Roseburia intestinalis_phage* |  |
|  |  |  | *Desulfitobacterium hafniense_phage* |  |
|  |  |  | *Acetivibrio cellulolyticus_phage* |  |
|  |  |  | *Ethanoligenens harbinense_phage* |  |
|  |  |  | *Corynebacterium argentoratense_phage* |  |
|  |  |  | *Corynebacterium aurimucosum_phage* |  |
|  |  |  | *Corynebacterium pseudogenitalium_phage* |  |
|  |  |  | *Mycobacterium avium_phage* |  |
|  |  |  | *Mycobacterium kansasii_phage* |  |
|  |  |  | *Desulfarculus baarsii_phage* |  |
|  |  |  | *Desulfovibrio sp. 6_1_46AFAA_phage* |  |
|  |  |  | *Citrobacter koseri_phage* |  |
|  |  |  | *Citrobacter rodentium_phage* |  |
|  |  |  | *Citrobacter youngae_phage* |  |
|  |  |  | *Edwardsiella piscicida_phage* |  |
|  |  |  | *Enterobacter asburiae_phage* |  |
|  |  |  | *Enterobacter mori_phage* |  |
|  |  |  | *Enterobacter sp. 638_phage* |  |
|  |  |  | *Enterobacter sp. R4-368_phage* |  |
|  |  |  | *Plautia stali symbiont_phage* |  |
|  |  |  | *Enterobacteriaceae_phage_n_n* |  |
|  |  |  | *Erwinia billingiae_phage* |  |
|  |  |  | *Escherichia sp. TW09231_phage* |  |
|  |  |  | *Klebsiella oxytoca_phage* |  |
|  |  |  | *Klebsiella variicola_phage* |  |
|  |  |  | *Morganella morganii_phage* |  |
|  |  |  | *Proteus penneri_phage* |  |
|  |  |  | *Rahnella sp. Y9602_phage* |  |
|  |  |  | *Raoultella ornithinolytica_phage* |  |
|  |  |  | *Lacinutrix sp. 5H-3-7-4_phage* |  |
|  |  |  | *Fusobacterium necrophorum_phage* |  |
|  |  |  | *Leptotrichia goodfellowii_phage* |  |
|  |  |  | *Carnobacterium maltaromaticum_phage* |  |
|  |  |  | *Lactobacillaceae_phage_u_u* |  |
|  |  |  | *Lactobacillus amylovorus_phage* |  |
|  |  |  | *Lactobacillus antri_phage* |  |
|  |  |  | *Lactobacillus buchneri_phage* |  |
|  |  |  | *Lactobacillus farciminis_phage* |  |
|  |  |  | *Lactobacillus paracasei_phage* |  |
|  |  |  | *Lactobacillus plantarum_phage* |  |
|  |  |  | *Enterococcus italicus_phage* |  |
|  |  |  | *Lactococcus_phage_u* |  |
|  |  |  | *Streptococcus ictaluri_phage* |  |
|  |  |  | *Streptococcus parauberis_phage* |  |
|  |  |  | *Streptococcus porcinus_phage* |  |
|  |  |  | *Legionella pneumophila_phage* |  |
|  |  |  | *Methylococcus capsulatus_phage* |  |
|  |  |  | *Gallibacterium anatis_phage* |  |
|  |  |  | *Acinetobacter johnsonii_phage* |  |
|  |  |  | *Acinetobacter nosocomialis_phage* |  |
|  |  |  | *Acinetobacter pittii_phage* |  |
|  |  |  | *Acinetobacter radioresistens_phage* |  |
|  |  |  | *Pseudomonas chlororaphis_phage* |  |
|  |  |  | *Pseudomonas coronafaciens_phage* |  |
|  |  |  | *Pseudomonas mendocina_phage* |  |
|  |  |  | *Pseudomonas parafulva_phage* |  |
|  |  |  | *Pseudomonas savastanoi_phage* |  |
|  |  |  | *Pseudomonas sp. M1_phage* |  |
|  |  |  | *Pseudomonas sp. TJI-51_phage* |  |
|  |  |  | *Pseudomonas syringae group genomosp. 3_phage* |  |
|  |  |  | *Acinetobacter baumannii_phage* |  |
|  |  |  | *Nitrobacter sp. Nb-311A_phage* |  |
|  |  |  | *Nitrobacter winogradskyi_phage* |  |
|  |  |  | *Rhodopseudomonas palustris_phage* |  |
|  |  |  | *Hyphomicrobium denitrificans_phage* |  |
|  |  |  | *Methylocystis sp. ATCC 49242_phage* |  |
|  |  |  | *Mesorhizobium sp. LNJC372A00_phage* |  |
|  |  |  | *Agrobacterium tumefaciens_phage* |  |
|  |  |  | *Rhizobium sp. IRBG74_phage* |  |
|  |  |  | *Paracoccus denitrificans_phage* |  |
|  |  |  | *Roseovarius sp. TM1035_phage* |  |
|  |  |  | *Acidaminococcus_phage_u* |  |
|  |  |  | *Novosphingobium pentaromativorans_phage* |  |
|  |  |  | *Sphingobium yanoikuyae_phage* |  |
|  |  |  | *Sphingopyxis alaskensis_phage* |  |
|  |  |  | *Sphingopyxis fribergensis_phage* |  |
|  |  |  | *Streptomyces collinus_phage* |  |
|  |  |  | *Streptomyces fulvissimus_phage* |  |
|  |  |  | *Streptomyces griseoaurantiacus_phage* |  |
|  |  |  | *Thermus parvatiensis_phage* |  |
|  |  |  | *Anaerococcus lactolyticus_phage* |  |
|  |  |  | *Anaerococcus_phage_u* |  |
|  |  |  | *Peptoniphilus lacrimalis_phage* |  |
|  |  |  | *Peptoniphilus sp. oral taxon 836_phage* |  |
|  |  |  | *Peptoniphilus_phage_u* |  |
|  |  |  | *Vibrio alginolyticus_phage* |  |
|  |  |  | *Vibrio ichthyoenteri_phage* |  |
|  |  |  | *Stenotrophomonas sp. SKA14_phage* |  |
|  |  |  | *Xanthomonas axonopodis_phage* |  |
|  |  |  | *Xanthomonas citri_phage* |  |
|  |  |  | *Xylella fastidiosa_phage* |  |
|  |  |  | *Polymorphum gilvum_phage* |  |
|  |  |  | *Escherichia coli_phage* |  |
|  |  |  | ***Treponema phage*** |  |
|  |  |  | ***Acinetobacter phage*** |  |
|  |  |  | ***Mannheimia phage*** |  |
|  |  |  | ***Pectobacterium phage*** |  |
|  |  |  | ***Phage TP*** |  |
|  |  |  | ***Salmonella phage*** |  |
|  |  |  | ***Haemophilus phage*** |  |
|  |  |  | ***P2likevirus_u*** |  |
|  |  |  | ***Streptococcus phage Cp-1*** |  |
|  |  |  | ***Enterobacteria phage HK022*** |  |
|  |  |  | ***Enterobacterial phage*** |  |
|  |  |  | ***Escherichia phage*** |  |
|  |  |  | ***Lambdalikevirus_u*** |  |
|  |  |  | ***Mannheimia phage*** |  |
|  |  |  | ***Streptococcus phage*** |  |
|  |  |  | ***Enterobacterial phage*** |  |
|  |  |  | ***Geobacillus virus E3*** |  |
|  |  |  | ***Lactobacillus phage JCL1032*** |  |
|  |  |  | ***Mycobacterium phage*** |  |
|  |  |  | ***Streptococcus phage PH15*** |  |
|  |  |  | ***Streptococcus phage*** |  |
|  |  |  | ***Skunalikevirus_u*** |  |
|  |  |  | ***Herpesviridae_u_u*** |  |
|  |  |  | ***Human herpesvirus 1*** |  |
|  |  |  | ***Simplexvirus_u*** |  |
|  |  |  | ***Torque teno virus*** |  |
|  |  |  | ***Pseudomonas phage Pf1*** |  |
|  |  |  | ***Betapapillomavirus 1*** |  |
|  |  |  | ***Betapapillomavirus 2*** |  |
|  |  |  | ***Gammapapillomavirus 13*** |  |
|  |  |  | ***Gammapapillomavirus 7*** |  |
|  |  |  | ***Gammapapillomavirus 8*** |  |
|  |  |  | ***Human papillomavirus 132-like viruses*** |  |
|  |  |  | ***Papillomaviridae_u_u*** |  |
|  |  |  | ***KI polyomavirus*** |  |
|  |  |  | ***Orf virus*** |  |
|  |  |  | ***Porcine type-C oncovirus*** |  |
|  |  |  | ***Human immunodeficiency virus*** |  |
|  |  |  | ***Citrus endogenous pararetrovirus*** |  |
|  |  |  | ***Escherichia phage*** |  |
|  |  |  | ***Geobacillus virus E2*** |  |
|  |  |  | ***Lactobacillus prophage Lj771*** |  |
|  |  |  | ***Leuconostoc phage*** |  |
|  |  |  | ***Staphylococcus phage tp310-1*** |  |
|  |  |  | ***Leuconostoc phage*** |  |

**(C)**

| **Presence** | | | | | | |
| --- | --- | --- | --- | --- | --- | --- |
| **High (>75%)** | **Medium-high** **(50**-**75%)** | | **Medium-low (25-50%)** | **Low (<25%)** | | **Singletons** |
| **9** | **7** | | **4** | **55** | | **17** |
| **75** | | | | | |
| Streptococcaceae_phage | **undefined** | Enterobacteriaceae_phage | | | Corynebacteriaceae_phage | Aeromonadaceae_phage |
| **Siphoviridae** | undefined | Carnobacteriaceae_phage | | | Erysipelotrichaceae_phage | Bacillales_phage_u |
| Pasteurellaceae_phage | Prevotellaceae_phage | Peptostreptococcaceae_phage | | | Spirochaetaceae_phage | Oxalobacteraceae_phage |
| Neisseriaceae_phage | **Podoviridae** | Campylobacteraceae_phage | | | Enterococcaceae_phage | Helicobacteraceae_phage |
| **Herpesviridae** | Fusobacteriaceae_phage |  | | | Porphyromonadaceae_phage | Clostridiaceae_phage |
| Veillonellaceae_phage | **u_u_Streptococcus phage** |  | | | Lactobacillaceae_phage | Brucellaceae_phage |
| **n_n_Streptococcus phage** | Lachnospiraceae_phage |  | | | Pseudomonadaceae_phage | Acetobacteraceae_phage |
| **Myoviridae** |  |  | | | Aerococcaceae_phage | Rhodospirillaceae_phage |
| **Retroviridae** |  |  | | | Moraxellaceae_phage | Streptomycetaceae_phage |
|  |  |  | | | Peptoniphilaceae_phage | **Caudovirales_u** |
|  |  |  | | | **n_n_Streptococcus phi-m46.1-like phage** | **Anelloviridae** |
|  |  |  | | | Actinomycetaceae_phage | **Inoviridae** |
|  |  |  | | | **n_n_Phage SK137** | **Polyomaviridae** |
| |  | | --- | |  |  | | | Cardiobacteriaceae_phage | **n_n_Geobacillus virus E2** |
|  |  |  | | | Atopobiaceae_phage | **n_n_Lactobacillus prophage Lj771** |
|  |  |  | | | Lactobacillales_phage_n | **n_n_Leuconostoc phage** |
|  |  |  | | | Bacillales_n_phage | **u_u_Leuconostoc phage** |
|  |  |  | | | Staphylococcaceae_phage |  |
|  |  |  | | | Lactobacillales_phage_u |  |
|  |  |  | | | **n_n_Streptococcus phage K13** |  |
|  |  |  | | | Bifidobacteriaceae_phage |  |
|  |  |  | | | **Papillomaviridae** |  |
|  |  |  | | | Leuconostocaceae_phage |  |
|  |  |  | | | Sphingomonadaceae_phage |  |
|  |  |  | | | Xanthomonadaceae_phage |  |
|  |  |  | | | **n_n_Streptococcus phage DCC1738** |  |
|  |  |  | | | Clostridiales_n_phage |  |
|  |  |  | | | Bacillaceae_phage |  |
|  |  |  | | | Bacteroidales_phage_u |  |
|  |  |  | | | Burkholderiaceae_phage |  |
|  |  |  | | | Clostridiales_phage_u |  |
|  |  |  | | | Flavobacteriaceae_phage |  |
|  |  |  | | | Rhodocyclaceae_phage |  |
|  |  |  | | | **Microviridae** |  |
|  |  |  | | | **Poxviridae** |  |
|  |  |  | | | **n_n_Fusobacterium phage** |  |
|  |  |  | | | **n_n_Neisseria meningitidis phage** |  |
|  |  |  | | | **n_n_Pseudomonas phage** |  |
|  |  |  | | | Aeromonadaceae_phage |  |
|  |  |  | | | Bacillales_phage_u |  |
|  |  |  | | | Oxalobacteraceae_phage |  |
|  |  |  | | | Helicobacteraceae_phage |  |
|  |  |  | | | Clostridiaceae_phage |  |
|  |  |  | | | Brucellaceae_phage |  |
|  |  |  | | | Acetobacteraceae_phage |  |
|  |  |  | | | Rhodospirillaceae_phage |  |
|  |  |  | | | Streptomycetaceae_phage |  |
|  |  |  | | | **Caudovirales_u** |  |
|  |  |  | | | **Anelloviridae** |  |
|  |  |  | | | **Inoviridae** |  |
|  |  |  | | | **Polyomaviridae** |  |
|  |  |  | | | **n_n_Geobacillus virus E2** |  |
|  |  |  | | | **n_n_Lactobacillus prophage Lj771** |  |
|  |  |  | | | **n_n_Leuconostoc phage** |  |
|  |  |  | | | **u_u_Leuconostoc phage** |  |

**(D)**

| **Presence** | | | | |
| --- | --- | --- | --- | --- |
| **High (>75%)** | **Medium-high (50-75%)** | **Medium-low (25-50%)** | **Low (<25%)** | **Singletons** |
| **7** | **17** | **30** | **247** | **101** |
| **301** | | | |
| *Streptococcus pneumoniae_phage* | *Neisseria gonorrhoeae_phage* | *Veillonella parvula_phage* | *Enterobacteriaceae_phage_u_u* | *Mobiluncus mulieris_phage* |
| *Streptococcus mitis_phage* | *Haemophilus haemolyticus_phage* | *Catonella morbi_phage* | *Streptococcus vestibularis_phage* | *Aeromonas hydrophila_phage* |
| *Streptococcus_phage_u* | ***Siphoviridae_n_u*** | *Streptococcus salivarius_phage* | ***Streptococcus phage*** | *Bacillus cereus_phage* |
| ***Human herpesvirus 7*** | ***Myoviridae_n_u*** | ***Human endogenous retrovirus*** | *Actinobacillus pleuropneumoniae_phage* | *Geobacillus sp. Y4.1MC1_phage* |
| ***Siphovirus contig89*** | *undefined* | *Neisseria lactamica_phage* | *Campylobacter concisus_phage* | *Bacillales_phage_u_u_u* |
| ***Streptococcus phage*** | ***undefined*** | *Neisseria_phage_u* | *Solobacterium moorei_phage* | *Staphylococcus xylosus_phage* |
| *Haemophilus influenzae_phage* | *Streptococcus infantis_phage* | *Veillonella dispar_phage* | *Streptococcus suis_phage* | *Prevotella dentalis_phage* |
|  | *Prevotella veroralis_phage* | ***Streptococcus phage*** | *Treponema socranskii_phage* | *Prevotella sp. oral taxon 472_phage* |
|  | *Streptococcus oralis_phage* | ***Siphoviridae_u_u*** | *Human endogenous retrovirus W* | *Bifidobacterium longum_phage* |
|  | *Haemophilus_phage_u* | *Streptococcus dysgalactiae_phage* | *Barnesiella viscericola_phage* | *Bifidobacterium pseudocatenulatum_phage* |
|  | ***Human endogenous retrovirus H*** | *Megasphaera micronuciformis_phage* | *Aggregatibacter aphrophilus_phage* | *Burkholderia multivorans_phage* |
|  | ***Streptococcus phage*** | *Granulicatella adiacens_phage* | *Haemophilus sputorum_phage* | *Ralstonia sp. 5_7_47FAA_phage* |
|  | ***Retroviridae_n_u*** | *Pasteurellaceae_phage_u_u* | *Prevotella pallens_phage* | *Janthinobacterium agaricidamnosum_phage* |
|  | *Fusobacterium nucleatum_phage* | *Selenomonas ruminantium_phage* | *Corynebacterium jeikeium_phage* | *Campylobacter rectus_phage* |
|  | ***Podoviridae_n_u*** | *Neisseria flavescens_phage* | *Neisseria polysaccharea_phage* | *Helicobacter pylori_phage* |
|  | *Streptococcus sanguinis_phage* | *Neisseria subflava_phage* | *Escherichia coli_phage* | *Clostridium bornimense_phage* |
|  | *Neisseria meningitidis_phage* | ***Hpunalikevirus_u*** | *Abiotrophia defectiva_phage* | *[Bacteroides] pectinophilus_phage* |
|  |  | ***Human endogenous retrovirus K*** | ***Sfi21dtunalikevirus_u*** | *[Clostridium] saccharolyticum_phage* |
|  |  | *Prevotella marshii_phage* | *Streptococcus sp. I-P16_phage* | *Lachnospiraceae bacterium 6_1_63FAA_phage* |
|  |  | ***Human herpesvirus 4*** | *Streptococcus phi-m46.1-like phage* | *[Eubacterium] rectale_phage* |
|  |  | *Veillonella atypica_phage* | *Filifactor alocis_phage* | *Citrobacter freundii_phage* |
|  |  | *Veillonella sp. 3_1_44_phage* | *Peptoclostridium difficile_phage* | *Cronobacter sakazakii_phage* |
|  |  | *Neisseria sicca_phage* | *Enterococcus faecium_phage* | *Enterobacter cloacae_phage* |
|  |  | *Fusobacterium periodonticum_phage* | *Streptococcus mutans_phage* | *Enterobacteriaceae_phage_n_n* |
|  |  | *Streptococcus pseudopneumoniae_phage* | *Selenomonas sp. oral taxon 137_phage* | *Escherichia_phage_n* |
|  |  | ***Human herpesvirus 6*** | ***Phage SK137*** | *Klebsiella oxytoca_phage* |
|  |  | *Aggregatibacter sp. oral taxon 458_phage* | *Peptostreptococcus stomatis_phage* | *Klebsiella sp. MS 92-3_phage* |
|  |  | *Haemophilus aegyptius_phage* | *Streptococcus constellatus_phage* | *Klebsiella_phage_u* |
|  |  | *Selenomonas sp. oral taxon 149_phage* | *[Haemophilus] parasuis_phage* | *Salmonella enterica_phage* |
|  |  | ***Streptococcus phage MM1*** | *Parvimonas micra_phage* | *Fusobacterium gonidiaformans_phage* |
|  |  |  | ***Listeria phage*** | *Lactobacillus brevis_phage* |
|  |  |  | ***Lactococcus phage*** | *Lactobacillus buchneri_phage* |
| |  | | --- | |  |  | *Cardiobacterium hominis_phage* | *Lactobacillus crispatus_phage* |
|  |  |  | *Streptococcus parasanguinis_phage* | *Lactobacillus farciminis_phage* |
|  |  |  | *Aggregatibacter_phage_u* | *Lactobacillus paracasei_phage* |
|  |  |  | *Haemophilus parainfluenzae_phage* | *Lactobacillus reuteri_phage* |
|  |  |  | *Lactobacillales_phage_n_n_n* | *Lactobacillus rhamnosus_phage* |
|  |  |  | *Neisseria bacilliformis_phage* | *Leuconostoc carnosum_phage* |
|  |  |  | *Aggregatibacter segnis_phage* | *Streptococcaceae_phage_u_u* |
|  |  |  | *Acinetobacter baumannii_phage* | *Streptococcus parauberis_phage* |
|  |  |  | ***Haemophilus phage*** | *Kingella denitrificans_phage* |
|  |  |  | ***Myoviridae_u_u*** | *Kingella kingae_phage* |
|  |  |  | ***Lactococcus phage*** | *Neisseriaceae_phage_u_u* |
|  |  |  | *Prevotella_phage_u* | *Acinetobacter calcoaceticus_phage* |
|  |  |  | *Campylobacter curvus_phage* | *Acinetobacter sp. ATCC 27244_phage* |
|  |  |  | *Oribacterium sinus_phage* | *Acinetobacter sp. P8-3-8_phage* |
|  |  |  | *Atopobium parvulum_phage* | *Pseudomonas chlororaphis_phage* |
|  |  |  | *Lactobacillales_phage_u_u_u* | *Pseudomonas fluorescens_phage* |
|  |  |  | *Lactococcus lactis_phage* | *Pseudomonas pseudoalcaligenes_phage* |
|  |  |  | *Streptococcus anginosus_phage* | *Pseudomonas stutzeri_phage* |
|  |  |  | *Neisseria mucosa_phage* | *Pseudomonas_phage_u* |
|  |  |  | *Pasteurella multocida_phage* | *Ochrobactrum anthropi_phage* |
|  |  |  | *Pseudomonas aeruginosa_phage* | *Komagataeibacter medellinensis_phage* |
|  |  |  | *Dialister invisus_phage* | *Azospirillum lipoferum_phage* |
|  |  |  | *Veillonella_phage_u* | *Selenomonas artemidis_phage* |
|  |  |  | *Cercopithecine herpesvirus 5* | *Selenomonas_phage_u* |
|  |  |  | ***Retroviridae_n_n*** | *Sphingobium yanoikuyae_phage* |
|  |  |  | ***Streptococcus phage K13*** | *Treponema denticola_phage* |
|  |  |  | *Mobiluncus curtisii_phage* | *Streptomyces scabiei_phage* |
|  |  |  | *Prevotella nigrescens_phage* | *Peptoniphilus duerdenii_phage* |
|  |  |  | *Corynebacterium matruchotii_phage* | *Peptoniphilus sp. oral taxon 375_phage* |
|  |  |  | *Klebsiella pneumoniae_phage* | *Stenotrophomonas maltophilia_phage* |
|  |  |  | *Fusobacterium_phage_u* | *Xanthomonas axonopodis_phage* |
|  |  |  | *Streptococcus sp. C150_phage* | ***Caudovirales_u_u_u*** |
|  |  |  | *Neisseria macacae_phage* | ***Acidithiobacillus phage*** |
|  |  |  | *Simonsiella muelleri_phage* | ***Pectobacterium phage*** |
|  |  |  | *Avibacterium paragallinarum_phage* | ***Haemophilus phage*** |
|  |  |  | *Haemophilus pittmaniae_phage* | ***P2likevirus_u*** |
|  |  |  | *Propionibacterium phage* | ***Enterobacteria phage*** |
|  |  |  | *Mobiluncus_phage_n* | ***Streptococcus phage Cp-1*** |
|  |  |  | *Gemella haemolysans_phage* | ***Enterobacteria phage*** |
|  |  |  | *[Clostridium] asparagiforme_phage* | ***Escherichia phage*** |
|  |  |  | *Lachnospiraceae bacterium oral taxon 500_phage* | ***Phietalikevirus_u*** |
|  |  |  | *Enterococcus faecalis_phage* | ***Streptococcus phage*** |
|  |  |  | *Lactobacillus salivarius_phage* | ***Geobacillus virus E3*** |
|  |  |  | *Streptococcus equi_phage* | ***Lactobacillus phage JCL1032*** |
|  |  |  | *Streptococcus macedonicus_phage* | ***Staphylococcus phage*** |
|  |  |  | *Streptococcus pyogenes_phage* | ***Streptococcus phage*** |
|  |  |  | *Kingella oralis_phage* | ***Skunalikevirus_u*** |
|  |  |  | *Neisseria sp. oral taxon 014_phage* | ***Herpesviridae_u_u*** |
|  |  |  | *Neisseria_phage_n* | ***Human herpesvirus 1*** |
|  |  |  | *Pseudomonas putida_phage* | ***Simplexvirus_u*** |
|  |  |  | *Veillonellaceae_phage_u_u* | ***Torque teno virus*** |
|  |  |  | ***Moraxella phage*** | ***Enterobacteria phage*** |
|  |  |  | ***Roseolovirus_u*** | ***Betapapillomavirus 1*** |
|  |  |  | ***Streptococcus phage DCC1738*** | ***Betapapillomavirus 2*** |
|  |  |  | *Staphylococcus aureus_phage* | ***Gammapapillomavirus 13*** |
|  |  |  | *Prevotella bivia_phage* | ***Gammapapillomavirus 7*** |
|  |  |  | *Lachnoanaerobaculum saburreum_phage* | ***Gammapapillomavirus 8*** |
|  |  |  | *Atopobium rimae_phage* | ***Human papillomavirus 132-like viruses*** |
|  |  |  | *Bulleidia extructa_phage* | ***Papillomaviridae_u_u*** |
|  |  |  | *Oenococcus oeni_phage* | ***KI polyomavirus*** |
|  |  |  | *Eikenella corrodens_phage* | ***Vaccinia virus*** |
|  |  |  | *Neisseria elongata_phage* | ***Orf virus*** |
|  |  |  | *Neisseria weaveri_phage* | ***Porcine type-C oncovirus*** |
|  |  |  | *Actinobacillus ureae_phage* | ***Human immunodeficiency virus*** |
|  |  |  | *Aggregatibacter actinomycetemcomitans_phage* | ***Citrus endogenous pararetrovirus*** |
|  |  |  | *Histophilus somni_phage* | ***Geobacillus virus E2*** |
|  |  |  | *Acinetobacter lwoffii_phage* | ***Lactobacillus prophage Lj771*** |
|  |  |  | *Megasphaera sp. UPII 135-E_phage* | ***Leuconostoc phage*** |
|  |  |  | *Selenomonas sputigena_phage* | ***Leuconostoc phage*** |
|  |  |  | *Veillonella sp. 6_1_27_phage* |  |
|  |  |  | *Sphingomonas sp. MM-1_phage* |  |
|  |  |  | ***Staphylococcus phage*** |  |
|  |  |  | ***Erwinia phage*** |  |
|  |  |  | ***Actinomyces phage*** |  |
|  |  |  | ***Streptococcus phage*** |  |
|  |  |  | *Gemella morbillorum_phage* |  |
|  |  |  | *Staphylococcus epidermidis_phage* |  |
|  |  |  | *Bacteroidales_phage_u_u_u* |  |
|  |  |  | *Prevotella sp. oral taxon 299_phage* |  |
|  |  |  | *Bifidobacterium sp. 12_1_47BFAA_phage* |  |
|  |  |  | *Gardnerella vaginalis_phage* |  |
|  |  |  | *Clostridiales bacterium 1_7_47FAA_phage* |  |
|  |  |  | *Clostridiales_phage_u_u_u* |  |
|  |  |  | *Lachnospiraceae_n_phage_u* |  |
|  |  |  | *Lachnospiraceae_phage_u_u* |  |
|  |  |  | *Peptostreptococcaceae_phage_u_u* |  |
|  |  |  | *Enterobacter hormaechei_phage* |  |
|  |  |  | *Escherichia_phage_u* |  |
|  |  |  | *Capnocytophaga sp. oral taxon 338_phage* |  |
|  |  |  | *Enterococcus_phage_u* |  |
|  |  |  | *Lactobacillus fermentum_phage* |  |
|  |  |  | *Lactobacillus gasseri_phage* |  |
|  |  |  | *Lactobacillus_phage_u* |  |
|  |  |  | *Streptococcus agalactiae_phage* |  |
|  |  |  | *Streptococcus gallolyticus_phage* |  |
|  |  |  | *Streptococcus sp. oral taxon 056_phage* |  |
|  |  |  | *Streptococcus sp. oral taxon 071_phage* |  |
|  |  |  | *Actinobacillus minor_phage* |  |
|  |  |  | *Mannheimia haemolytica_phage* |  |
|  |  |  | *Acinetobacter_phage_u* |  |
|  |  |  | *Azospira oryzae_phage* |  |
|  |  |  | *Selenomonas noxia_phage* |  |
|  |  |  | *Xanthomonas campestris_phage* |  |
|  |  |  | ***Sfi1unalikevirus_u*** |  |
|  |  |  | ***Lactobacillus phage*** |  |
|  |  |  | ***Streptococcus phage Dp-1*** |  |
|  |  |  | ***Streptococcus phage PH10*** |  |
|  |  |  | ***Streptococcus phage SM1*** |  |
|  |  |  | ***Enterobacteria phage*** |  |
|  |  |  | ***Human papillomavirus*** |  |
|  |  |  | ***Papillomaviridae_n_n*** |  |
|  |  |  | ***Fusobacterium phage*** |  |
|  |  |  | ***Neisseria meningitidis phage*** |  |
|  |  |  | ***Pseudomonas phage*** |  |
|  |  |  | *Mobiluncus mulieris_phage* |  |
|  |  |  | *Aeromonas hydrophila_phage* |  |
|  |  |  | *Bacillus cereus_phage* |  |
|  |  |  | *Geobacillus sp. Y4.1MC1_phage* |  |
|  |  |  | *Bacillales_phage_u_u_u* |  |
|  |  |  | *Staphylococcus xylosus_phage* |  |
|  |  |  | *Prevotella dentalis_phage* |  |
|  |  |  | *Prevotella sp. oral taxon 472_phage* |  |
|  |  |  | *Bifidobacterium longum_phage* |  |
|  |  |  | *Bifidobacterium pseudocatenulatum_phage* |  |
|  |  |  | *Burkholderia multivorans_phage* |  |
|  |  |  | *Ralstonia sp. 5_7_47FAA_phage* |  |
|  |  |  | *Janthinobacterium agaricidamnosum_phage* |  |
|  |  |  | *Campylobacter rectus_phage* |  |
|  |  |  | *Helicobacter pylori_phage* |  |
|  |  |  | *Clostridium bornimense_phage* |  |
|  |  |  | *[Bacteroides] pectinophilus_phage* |  |
|  |  |  | *[Clostridium] saccharolyticum_phage* |  |
|  |  |  | *Lachnospiraceae bacterium 6_1_63FAA_phage* |  |
|  |  |  | *[Eubacterium] rectale_phage* |  |
|  |  |  | *Citrobacter freundii_phage* |  |
|  |  |  | *Cronobacter sakazakii_phage* |  |
|  |  |  | *Enterobacter cloacae_phage* |  |
|  |  |  | *Enterobacteriaceae_phage_n_n* |  |
|  |  |  | *Escherichia_phage_n* |  |
|  |  |  | *Klebsiella oxytoca_phage* |  |
|  |  |  | *Klebsiella sp. MS 92-3_phage* |  |
|  |  |  | *Klebsiella_phage_u* |  |
|  |  |  | *Salmonella enterica_phage* |  |
|  |  |  | *Fusobacterium gonidiaformans_phage* |  |
|  |  |  | *Lactobacillus brevis_phage* |  |
|  |  |  | *Lactobacillus buchneri_phage* |  |
|  |  |  | *Lactobacillus crispatus_phage* |  |
|  |  |  | *Lactobacillus farciminis_phage* |  |
|  |  |  | *Lactobacillus paracasei_phage* |  |
|  |  |  | *Lactobacillus reuteri_phage* |  |
|  |  |  | *Lactobacillus rhamnosus_phage* |  |
|  |  |  | *Leuconostoc carnosum_phage* |  |
|  |  |  | *Streptococcaceae_phage_u_u* |  |
|  |  |  | *Streptococcus parauberis_phage* |  |
|  |  |  | *Kingella denitrificans_phage* |  |
|  |  |  | *Kingella kingae_phage* |  |
|  |  |  | *Neisseriaceae_phage_u_u* |  |
|  |  |  | *Acinetobacter calcoaceticus_phage* |  |
|  |  |  | *Acinetobacter sp. ATCC 27244_phage* |  |
|  |  |  | *Acinetobacter sp. P8-3-8_phage* |  |
|  |  |  | *Pseudomonas chlororaphis_phage* |  |
|  |  |  | *Pseudomonas fluorescens_phage* |  |
|  |  |  | *Pseudomonas pseudoalcaligenes_phage* |  |
|  |  |  | *Pseudomonas stutzeri_phage* |  |
|  |  |  | *Pseudomonas_phage_u* |  |
|  |  |  | *Ochrobactrum anthropi_phage* |  |
|  |  |  | *Komagataeibacter medellinensis_phage* |  |
|  |  |  | *Azospirillum lipoferum_phage* |  |
|  |  |  | *Selenomonas artemidis_phage* |  |
|  |  |  | *Selenomonas_phage_u* |  |
|  |  |  | *Sphingobium yanoikuyae_phage* |  |
|  |  |  | *Treponema denticola_phage* |  |
|  |  |  | *Streptomyces scabiei_phage* |  |
|  |  |  | *Peptoniphilus duerdenii_phage* |  |
|  |  |  | *Peptoniphilus sp. oral taxon 375_phage* |  |
|  |  |  | *Stenotrophomonas maltophilia_phage* |  |
|  |  |  | *Xanthomonas axonopodis_phage* |  |
|  |  |  | ***Caudovirales_u_u_u*** |  |
|  |  |  | ***Acidithiobacillus phage*** |  |
|  |  |  | ***Pectobacterium phage*** |  |
|  |  |  | ***Haemophilus phage*** |  |
|  |  |  | ***P2likevirus_u*** |  |
|  |  |  | ***Enterobacteria phage*** |  |
|  |  |  | ***Streptococcus phage Cp-1*** |  |
|  |  |  | ***Enterobacteria phage*** |  |
|  |  |  | ***Escherichia phage*** |  |
|  |  |  | ***Phietalikevirus_u*** |  |
|  |  |  | ***Streptococcus phage*** |  |
|  |  |  | ***Geobacillus virus E3*** |  |
|  |  |  | ***Lactobacillus phage JCL1032*** |  |
|  |  |  | ***Staphylococcus phage*** |  |
|  |  |  | ***Streptococcus phage*** |  |
|  |  |  | ***Skunalikevirus_u*** |  |
|  |  |  | ***Herpesviridae_u_u*** |  |
|  |  |  | ***Human herpesvirus 1*** |  |
|  |  |  | ***Simplexvirus_u*** |  |
|  |  |  | ***Torque teno virus*** |  |
|  |  |  | ***Enterobacteria phage*** |  |
|  |  |  | ***Betapapillomavirus 1*** |  |
|  |  |  | ***Betapapillomavirus 2*** |  |
|  |  |  | ***Gammapapillomavirus 13*** |  |
|  |  |  | ***Gammapapillomavirus 7*** |  |
|  |  |  | ***Gammapapillomavirus 8*** |  |
|  |  |  | ***Human papillomavirus 132-like viruses*** |  |
|  |  |  | ***Papillomaviridae_u_u*** |  |
|  |  |  | ***KI polyomavirus*** |  |
|  |  |  | ***Vaccinia virus*** |  |
|  |  |  | ***Orf virus*** |  |
|  |  |  | ***Porcine type-C oncovirus*** |  |
|  |  |  | ***Human immunodeficiency virus*** |  |
|  |  |  | ***Citrus endogenous pararetrovirus*** |  |
|  |  |  | ***Geobacillus virus E2*** |  |
|  |  |  | ***Lactobacillus prophage Lj771*** |  |
|  |  |  | ***Leuconostoc phage*** |  |
|  |  |  | ***Leuconostoc phage*** |  |
