# Supplementary figures and images for: The analysis of the oral DNA virome reveals which viruses are widespread and rare among healthy young adults in Valencia (Spain)
Source: PLoS One. 2018 Feb 8;13(2):e0191867. doi: 10.1371/journal.pone.0191867 (PMC5805259; doi:10.1371/journal.pone.0191867)

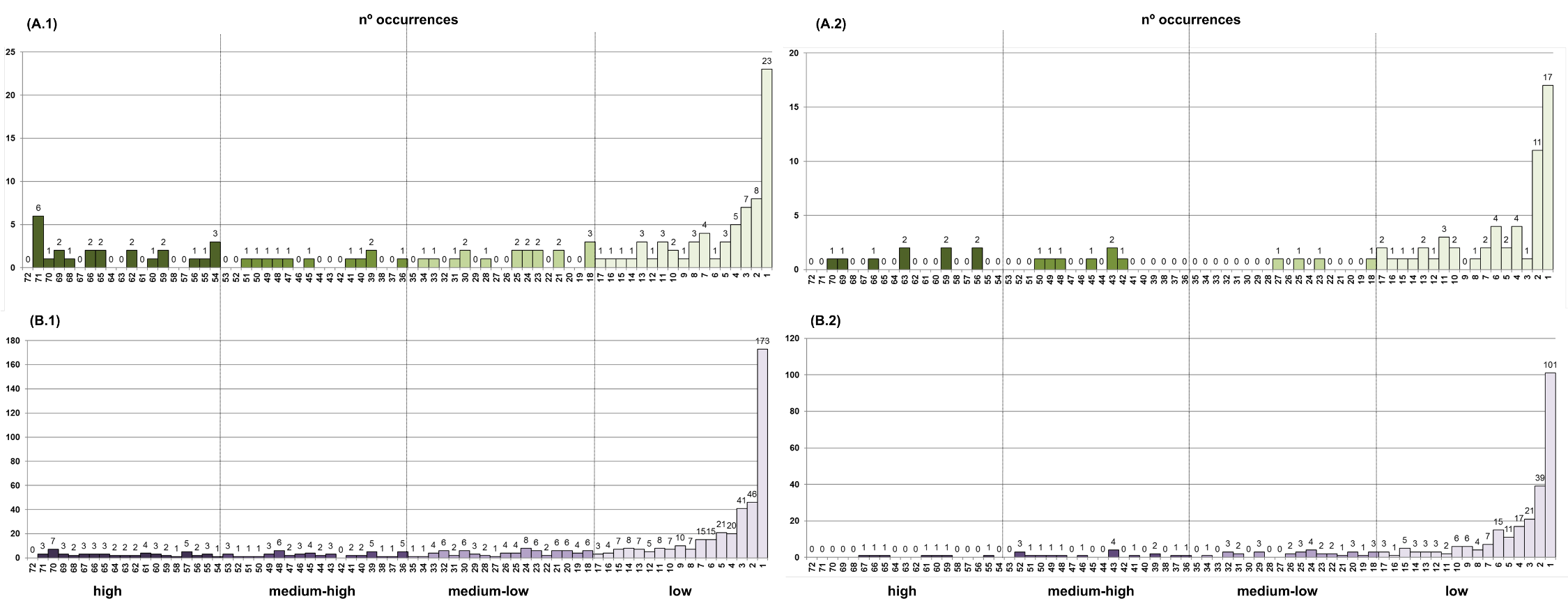

Supplement: S1 Fig — Number of occurrences for reads with homology to viruses and bacteria is displayed at family (A.1) and species level (A.2); whereas for reads with homology to viruses but without homology to bacteria is displayed at family (B.1) and species level (B.2). The four categories described (high, medium-high, medium-low and low) are shown. Green spectrum indicates family taxonomic level and purple spectrum indicates species level. (TIF) [file pone.0191867.s005.tif]

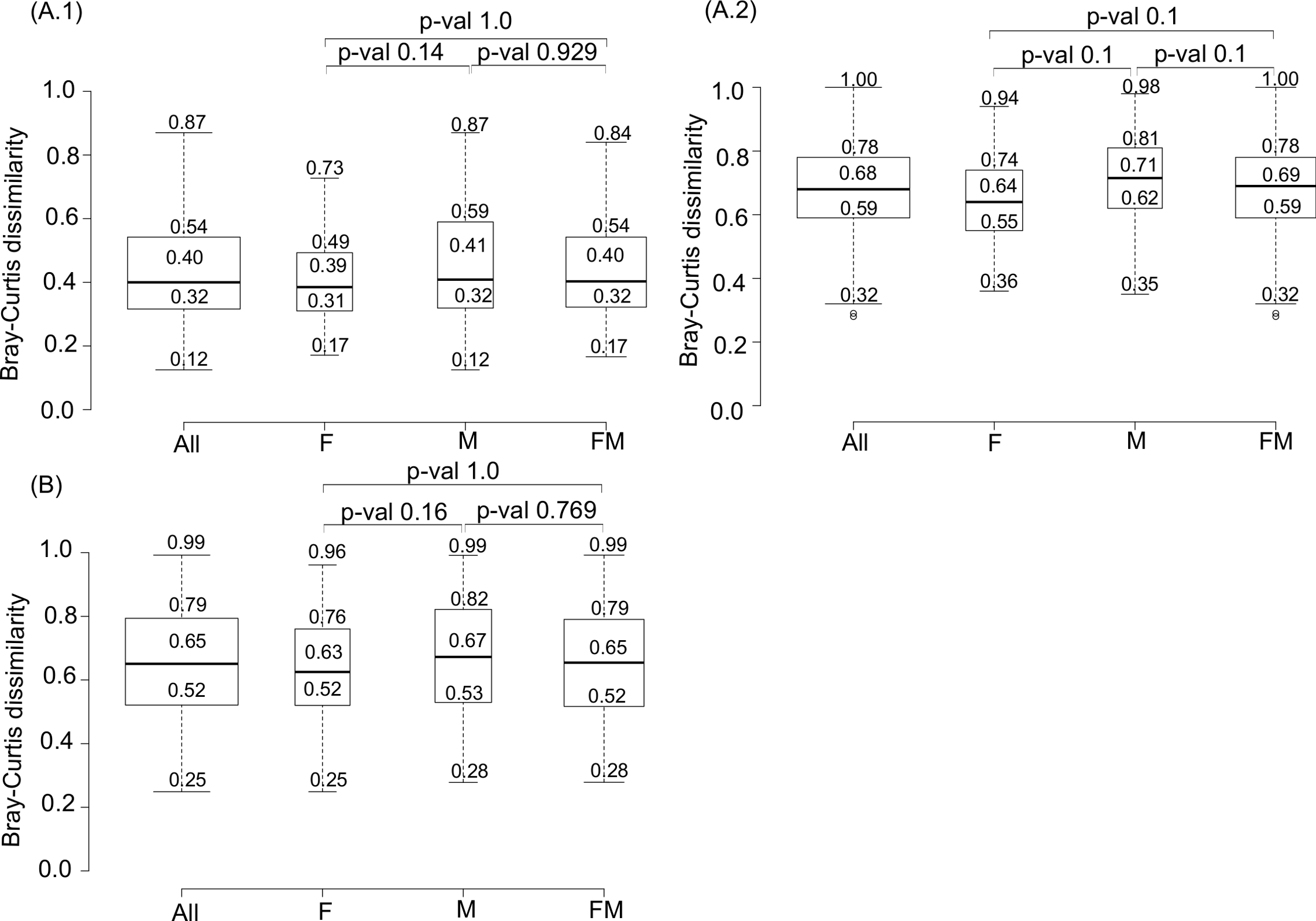

Supplement: S2 Fig — The maximum, 3rd quartile, median, 1st quartile and minimum values are depicted for each box and whisker. Outlier are also plotted. F: females; M males; FM: females-males. The statistical significance, based on Monte Carlo (nonparametric) tests with Bonferroni corrections, is also reported for the comparisons among sexes. (TIF) [file pone.0191867.s006.tif]
